# Synthesis, molecular modelling and anticancer evaluation of new pyrrolo[1,2-*b*]pyridazine and pyrrolo[2,1-*a*]phthalazine derivatives

**DOI:** 10.1080/14756366.2018.1550085

**Published:** 2019-01-03

**Authors:** Lacramioara Popovici, Roxana-Maria Amarandi, Ionel I. Mangalagiu, Violeta Mangalagiu, Ramona Danac

**Affiliations:** a Faculty of Chemistry, Alexandru Ioan Cuza University of Iasi, Iasi, Romania;; b CERNESIM Research Centre, Alexandru Ioan Cuza University of Iasi, Iasi, Romania

**Keywords:** Anticancer, phenstatin, pyrrolo[1,2-*b*]pyridazine, pyrrolo[2,1-*a*]phthalazine, 3 + 2 dipolar cycloaddition, docking, N-heterocycles

## Abstract

Two new series of heterocyclic derivatives with potential anticancer activity, in which a pyrrolo[1,2-*b*]pyridazine or a pyrrolo[2,1-*a*]phthalazine moiety was introduced in place of the 3′-hydroxy-4′-methoxyphenyl ring of phenstatin have been synthesised and their structure-activity relationship (SAR) was studied. Fourteen of the new compounds were evaluated for their *in vitro* cytotoxic activity by National Cancer Institute (NCI) against 60 human tumour cell lines panel. The best five compounds in terms of *in vitro* growth inhibition were screened in the second stage five dose-response studies, three of them showing a very good antiproliferative activity with GI_50_<100 nM on several cell lines including colon, ovarian, renal, prostate, brain and breast cancer, melanoma and leukemia. Docking experiments on the biologically active compounds showed a good compatibility with the colchicine binding site of tubulin.

## Introduction

Considerable efforts have been focussed in the past decades, on the design and development of new antiproliferative drugs with improved efficiency, limited toxicity, cost-effectiveness, which are synchronously less prone to develop multidrug resistance^1–3^. Among the variety of targets used in this huge anticancer fight, tubulin targeting appears to be a key focus in cancer treatment, the research in this field remaining very active in past years^4–7^. After the success of Colchicine^8^, combretastatin A-4^9^, vincristine or vinblastine^10^ as anticancer drugs acting by inhibiting tubulin polymerisation, research efforts focused on developing new colchicine binding site inhibitors with improved pharmacological profiles^4^^,^^11^.

One of the simplest known structures synthesised and tested as an anticancer agent in the past years is phenstatin^12^^,^^13^ which stand as one of the most potent tubulin polymerisation inhibitors by binding to the colchicine site of the tubulin and thus, interfering with the equilibrium dynamics associated with the cell division^14^^,^^15^. Because of its biological properties and structural simplicity, phenstatin continues to be a lead compound for rational design in anticancer therapy, the recent literature being plentiful of such phenstatin analogues^16–18^.

Pyrrolo-fused derivatives comprise a class of biologically active heterocyclic compounds which can serve as promising scaffolds for the development of anticancer, antimicrobial, antiviral, antimalarial, antitubercular, anti-inflammatory, and enzyme inhibiting drugs^19^. Among the fused pyrrolo-heterocyclic compounds, pyrrolo[1,2-*b*]pyridazines and its condensed pyrrolo[2,1-*a*]phthalazine system are compounds well known for their strong luminescence^20^^,^^21^ and photochromic properties^22^, and at the same time are promising in the field of drug design^23^^,^^24^, some derivatives being reported to have antimicrobial^25^^,^^26^, antifungal^25^ or anticancer effects^27^^,^^28^, or to act as acyl CoA:diacylglycerol acyltransferase (DGAT1) inhibitors^24^, JAK inhibitors^29^, HER-2 tyrosine kinase inhibitors^30^, IRAK4 inhibitors^31^, or MEK inhibitors^32^.

The replacement of one of the substituted phenyl ring of phenstatin with pyrrolo-fused heterocycles has been a major focus in rational drug design in the recent years, as there are several reported biological active phenstatin analogues containing an indole ring^5^^,^^19^, an indolizine ring^33^, or a pyrrolo[2,3-*d*]pyrimidine ring^34^. However, to our knowledge, there are no reported analogues of phenstatin with pyrrolo[1,2-*b*]pyridazine or pyrrolo[2,1-*a*]phthalazine scaffolds, respectively.

**Figure 1:**
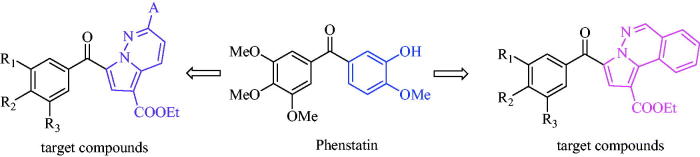
The structures of phenstatin and the target compounds.

With the aim of exploring new potential antitumour scaffolds, the target compounds described in this paper possess a pyrrolo[1,2-b]pyridazin-7-yl or a pyrrolo[2,1-a]phthalazin-3-yl moiety in place of the 3′-hydroxy-4′-methoxyphenyl ring of phenstatin. In order to establish structure-activity relationships (SARs), we extended our structural modifications by introducing different substituents at position 2 of the pyrrolo[1,2-b]pyridazine unit, including methyl or 4-substituted phenyl rings (4-chlorophenyl, 4-bromophenyl or *p*-tolyl). At the same time, the trimethoxyphenyl ring of phenstatin was replaced either by 3,5-dihydroxyphenyl, 3,4-dimethoxyphenyl or 4-bromophenyl (Figure 1).

## Materials and methods

### Chemistry

All commercially available reagents and solvents employed were used without further purification. Melting points were recorded on an A. Krüss Optronic Melting Point Meter KSPI and are uncorrected. Proton and carbon nuclear magnetic resonance (δ_H_, δ_C_) spectra were recorded on a DRX-500 Bruker or a Bruker Avance 400 DRX spectrometers. The following abbreviations were used to designate chemical shift multiplicities: s: singlet, d: doublet, t: triplet, q: quartet, m: multiplet, bs: broad singlet, as: apparent singlet. All chemical shifts are quoted on the δ-scale in ppm. Coupling constants are given in Hz. IR spectra were recorded on a FTIR Shimadzu or Jasco 660 plus FTIR spectrophotometer. Analyses indicated by the symbols of the elements or functions were within ±0.4% of the theoretical values. Thin layer chromatography (TLC) was carried out on Merck silica gel 60F_254_ plates. Visualisation of the plates was achieved using a UV lamp (λ_max_ = 254 or 365 nm).

Compounds 7d, 7h, 7l, 8d, 10a, and 10d were previously reported^35–39^.

#### General procedure for the synthesis of monoquaternary salts 7 and 10

1 mmol of heterocycle (pyridazine **1**, 3-methylpyridazine **2**, 3–(4-chlorophenyl)pyridazine) **3**, 3–(4-bromophenyl)pyridazine **4**, 3-(*p*-tolyl)pyridazine **5** or phthalazine **9** was dissolved in 7 ml acetone (for compounds **1–5**) or acetonitrile for compound **9**. Then 1.1 mmol of reactive halide (2-bromo-1–(3,4,5-trimethoxyphenyl)ethanone **6a**, 2-bromo-1–(3,5-dimethoxyphenyl) ethanone **6 b**, 2-bromo-1–(3,4-dimethoxyphenyl) ethanone **6c** or 2-bromo-1–(4-bromophenyl) ethanone **6d**) was added and the resulted mixture was stirred overnight at room temperature (to obtain compounds **7**) or reflux (for synthesis of compounds **10**). The reaction mixture was cooled and the formed precipitate was filtered and washed with diethyl ether to give the desired product which was used in the next reaction without any further purification. In case of salts **7e–g**, the resulting salts have not crystallised; for these compounds, the solvent was removed under vacuum, and the resulting liquid was used in the next step.

#### General procedure for preparation of compounds 8a–t and 11a–d

The cycloimmonium salt (**7a–d** or **10a–d)** (1 mmol) and ethyl propiolate (1.1 mmol) were added to 10 ml of anhydrous acetone and the obtained suspension was stirred at room temperature. Then, a solution of triethylamine (TEA) (3 mmol, 3 equiv.) in anhydrous acetone (3 ml) was added drop-wise over 1 h (magnetic stirring) and the resulting mixture was then stirred overnight at room temperature. Water (10 ml) was added and the formed solid was collected by filtration to give a powder which was washed with 5 ml methanol. The product was crystallised from dichloromethane/methanol (1:1, v/v).

*1–(2-Oxo-2–(3,4,5-trimethoxyphenyl)ethyl)pyridazin-1-ium bromide****7a***. Beige powder, Yield: 61%; m.p. 152–153 °C; IR (KBr, cm^−1^): 1672, 1583, 1416, 1319, 1165, 1128. ^1^H NMR (500 MHz DMSO-d_6_) *δ* 3.80 (s, 3H, OMe), 3.90 (s, 6H, 2 × OMe), 6.82 (s, 2H, H7), 7.39 (s, 2H, H10, H14), 8.76 (t, *J* =  6.0 Hz, 1H, H4), 8.90 (t, *J* =  6.0 Hz, 1H, H5), 9.74 (d, *J* =  3.5 Hz, 1H, H3), 9.74 (d, *J* =  5.0 Hz, 1H, H6). ^13 ^C NMR (125 MHz DMSO-d_6_) *δ* 56.4 (2 × OMe), 60.4 (OMe), 70.3 (C7), 106.2 (C10, C14), 136.0 (C5), 137.5 (C4), 143.3 (C12), 151.9 (C6), 153.1 (C11, C13), 154.7 (C3), 189.3 (C8). Anal. calcd. for C_15_H_17_BrN_2_O_4_: C, 48.80; H, 4.64; N, 7.59%. Found: C, 48.79; H, 4.55; N, 7.62%.

*1–(2-Oxo-2–(3,5-dimethoxyphenyl)ethyl)pyridazin-1-ium bromide****7b***. Beige powder, Yield: 60%; m.p. 196–199 °C; IR (KBr, cm^−1^): 1684, 1586, 1438, 1334, 1209, 1157, 1058. ^1^H NMR (500 MHz DMSO-d_6_): *δ* 3.85 (s, 6H, 2 × OMe), 6.76 (s, 2H, H7), 6.94 (t, *J* =  2.0 Hz, 1H, H12), 7.21 (d, *J* =  2.5 Hz, 2H, H10, H14), 8.75 (dd, *J* =  8.0; 4.5 Hz, 1H, H4), 8.88 (m, 1H, H5), 9.74 (d, *J* =  5.5 Hz, 1H, H3), 9.90 (d, *J* =  5.5 Hz, 1H, H6). ^13 ^C NMR (125 MHz DMSO-d_6_): *δ* 55.8 (2 × OMe), 70.4 (C7), 106.3 (C10, C14), 106.5 (C12), 135.2 (C9), 136.0 (C5), 137.6 (C4), 151.9 (C6), 154.7 (C3), 160.9 (C11, C13), 190.2 (C8). Anal. calcd. for C_14_H_15_BrN_2_O_3_: C, 49.57; H, 4.46; N, 8.26%. Found: C, 49.59; H, 4.42; N, 8.32%.

*1–(2-Oxo-2–(3,4-dimethoxyphenyl)ethyl)pyridazin-1-ium bromide****7c***. Beige solid, Yield: 60%; m.p. 123–124 °C; IR (KBr, cm^−1^): 2979, 1677, 1586, 1518, 1294, 1205, 1168. ^1^H NMR (500 MHz DMSO-d_6_): δ 3.85 (s, 3H, OMe), 3.90 (s, 3H, OMe), 6.79 (s, 2H, H7), 7.22 (d, *J* =  8.5 Hz, 1H, H13), 7.53 (d, *J* =  2.0 Hz, 1H, H10), 7.81 (dd, *J* =  8.5; 2.0 Hz, 1H, H14), 8.77 (m, 1H, H4), 8.91 (m, 1H, H5), 9.75 (dd, *J* =  5.0; 1.0 Hz, 1H, H3), 9.97 (d, *J* =  6.0 Hz, 1H, H6). ^13 ^C NMR (125 MHz DMSO-d_6_): *δ* 55.8 (OMe), 56.1 (OMe), 70.1 (C7), 110.4 (C10), 111.3 (C14), 123.7 (C13), 126.0 (C9), 136.0 (C5), 137.5 (C4), 151.9 (C6), 154.7 (C3), 148.9 (C11), 154.5 (C12), 188.7 (C8). Anal. calcd. for C_14_H_15_BrN_2_O_3_: C, 49.57; H, 4.46; N, 8.26%. Found: C, 49.55; H, 4.44; N, 8.30%.

*1–(2-Oxo-2–(4-bromophenyl)ethyl)pyridazin-1-ium bromide****7d***. White solid, Yield: 66%; m.p. 235–237 °C; IR (KBr, cm^−1^): 3017, 2976, 1695, 1580, 1439, 1394, 1229, 976, 822. ^1^H NMR (400 MHz DMSO-d_6_): δ 6.75 (s, 2H, H7), 7.90 (d, *J* =  8.4 Hz, 2H, H11, H13), 8.03 (d, *J* =  8.4 Hz, 2H, H10, H14), 8.78 (m, 1H, H4), 8.92 (m, 1H, H5), 9.76 (dd, *J* =  6.0; 0.8 Hz, 1H, H3), 9.99 (d, *J* =  6.0 Hz, 1H, H6). ^13 ^C NMR (100 MHz DMSO-d_6_): *δ* 70.2 (C7), 129.1 (C12), 130.4 (C10, C14), 132.2 (C11, C13), 135.4 (C9), 136.0 (C5), 137.5 (C4), 151.8 (C6), 154.7 (C3), 189.8 (C8). Anal. calcd. for C_12_H_10_Br_2_N_2_O: C, 40.26; H, 2.82; N, 7.82%. Found: C, 40.25; H, 2.79; N, 7.84%.

*3-Methyl-1–(2-oxo-2–(3,4,5-trimethoxyphenyl)ethyl)pyridazin-1-ium bromide****7e***. Liquid, Yield: 50%; IR (cm^−1^): 3069, 2943, 1687, 1586, 1417, 1332, 1126, 1050, 921. ^1^H NMR (500 MHz DMSO-d_6_) δ 2.81 (s, 3H, Me), 3.80 (s, 3H, OMe), 3.89 (s, 6H, 2 × OMe), 6.76 (s, 2H, H7), 7.38 (s, 2H, H10, H14), 8.65 (d, *J* =  8.5 Hz, 1H, H4), 8.77 (dd, *J* =  8.5; 6.0 Hz, 1H, H5), 9.80 (d, *J* =  6.0 Hz, 1H, H6). ^13 ^C NMR (125 MHz DMSO-d_6_): *δ* 21.6 (Me), 56.4 (2 × OMe), 60.4 (OMe), 70.1 (C7), 106.2 (C10, C14), 128.5 (C9), 135.0 (C5), 138.4 (C4), 143.3 (C12), 149.7 (C6), 153.1 (2 x C11), 164.9 (C3), 189.3 (C8).

*3-Methyl-1–(2-oxo-2–(3,5-dimethoxyphenyl)ethyl)pyridazin-1-ium bromide****7f***. Liquid, Yield: 50%; IR (cm^−1^): 3069, 2943, 1687, 1586, 1417, 1332, 1164, 1050, 921. ^1^H NMR (500 MHz DMSO-d_6_): δ 2.90 (s, 3H, Me), 3.84 (s, 6H, 2 × OMe), 6.63 (s, 2H, H7), 6.90 (t, *J* =  2.0 Hz, 1H, H12), 7.20 (d, *J* =  2.5 Hz, 2H, H10, H14), 8.67 (d, *J* =  8.5 Hz, 1H, H4), 8.78 (dd, *J* =  8.5; 5.5 Hz, 1H, H5), 9.87 (d, *J* =  5.5 Hz, 1H, H6). ^13 ^C NMR (125 MHz DMSO-d_6_) *δ* 21.6 (Me), 55.9 (2 × OMe), 70.3 (C7), 106.3 (C10, C14), 106.6 (C12); 135.0 (C9), 135.2 (C5), 138.4 (C4), 149.6 (C6), 160.8 (C11, C13), 164.8 (C3), 190.2 (C8).

*3-Methyl-1–(2-oxo-2–(3,4-dimethoxyphenyl)ethyl)pyridazin-1-ium bromide****7g***. Liquid; Yield: 51%; IR (cm^−1^): 2969, 1682, 1586, 1517, 1270, 1019, 806. ^1^H NMR (DMSO-d_6_, 500 MHz) δ 2.80 (s, 3H, Me), 3.84 (s, 3H, OMe), 3.84 (s, 3H, OMe), 6.78 (s, 2H, H7), 7.18 (d, *J* =  8.5 Hz, 1H, H12), 7.51 (d, *J* =  2.0 Hz, 1H, H10), 7.79 (dd, *J* =  8.5; 2.0 Hz, 1H, H14), 8.68 (1H, d, *J* =  8.5 Hz, H4), 8.80 (1H, dd, *J* =  8.5; 5.5 Hz, H5), 9.93 (d, *J* =  5.5 Hz, 1H, H6). ^13 ^C NMR (125 MHz DMSO-d_6_): *δ* = 21.7 (Me), 55.8 (OMe), 56.0 (OMe), 70.0 (C_7_), 110.5 (C_10_), 111.4 (C_14_), 123.8 (C_13_), 126.1 (C_9_), 135.0 (C_5_), 138.3 (C_4_), 149.7 (C_6_), 148.9 (C_11_), 154.5 (C_12_), 164.8 (C_3_), 188.7 (C_8_).

*3-Methyl-1–(2-oxo-2–(4-bromophenyl)ethyl)pyridazin-1-ium bromide****7h***. Beige solid, Yield: 70%; m.p. 216–218 °C; IR (KBr, cm^−1^): 3013, 2976, 1692, 1586, 1468, 1231, 1072, 988, 828. ^1^H NMR (500 MHz DMSO-d_6_) δ 2.81 (s, 3H, Me), 6.74 (s, 2H, H7), 7.89 (d, *J* =  8.5 Hz, 2H, H11, H13), 8.02 (d, *J* =  8.5 Hz, 2H, H10, H14), 8.66 (d, *J* =  8.5 Hz, 1H, H4), 8.78 (dd, *J* =  8.5; 6.0 Hz, 1H, H5), 9.82 (d, *J* =  5.5 Hz, 1H, H6). ^13 ^C NMR (125 MHz DMSO-d_6_) *δ* 21.6 (Me), 70.0 (C7), 129.1 (C12); 130.4 (C10, C14), 132.3 (C11, C13), 132.4 (C9), 135.0 (C5), 138.4 (C4), 149.7 (C6), 164.8 (C3), 189.8 (C8). Anal. calcd. for C_13_H_12_Br_2_N_2_O: C, 41.97; H, 3.25; N, 7.53%. Found: C, 42.00; H, 3.18; N, 7.55%.

*3–(4-Chlorophenyl)-1–(2-oxo-2–(3,4,5-trimethoxyphenyl)ethyl)pyridazin-1-ium bromide****7i***. Beige **s**olid, Yield 74%; m.p. 186–188 °C; IR (KBr, cm^−1^): 3059, 2932, 1682, 1599, 1450, 1343, 1132. ^1^H NMR (500 MHz DMSO-d_6_) δ 3.81 (s, 3H, OMe), 3.90 (s, 6H, 2 × OMe), 6.91 (s, 2H, H7), 7.42 (s, 2H, H10, H14), 7.76 (d, *J* =  7.5 Hz, 2H, H17, H19), 8.25 (d, *J* =  8.0 Hz, 2H, H16, H20), 8.96 (dd, *J* =  9.0; 4.5 Hz, 1H, H5), 9.33 (d, *J* =  9.0 Hz, 1H, H4), 9.93 (d, *J* =  4.5 Hz, 1H, H6). ^13 ^C NMR (125 MHz DMSO-d_6_) *δ* 56.3 (2 × OMe), 60.3 (OMe), 70.5 (C7), 106.2 (C10, C14), 128.4 (C9), 129.6 (C16, C20, C17, C19), 130.5 (C15), 134.7 (C4), 136.3 (C5), 137.7 (C18), 143.2 (C12), 150.0 (C6), 153.0 (C11, C13), 159.9 (C3), 189.1 (C8). Anal. calcd. for C_21_H_20_BrClN_2_O_4_: C, 52.57; H, 4.20; N, 5.84%. Found: C, 52.55; H, 4.18; N, 5.87%.

*3–(4-Chlorophenyl)-1–(2-oxo-2–(3,5-dimethoxyphenyl)ethyl)pyridazin-1-ium bromide****7j***. Beige solid, Yield: 75%; m.p. 220–222 °C; IR (KBr, cm^−1^): 3034, 2980, 1697, 1599, 1456, 1356, 1204, 1153, 841. ^1^H NMR (500 MHz DMSO-d_6_) δ 3.86 (s, 6H, 2 × OMe), 6.83 (s, 2H, H7), 6.95 (as, 1H, H12), 7.24 (as, 2H, H10, H14), 7.77 (d, *J* =  8.5 Hz, 2H, H17, H19), 8.25 (d, *J* =  8.5 Hz, 2H, H16, H20), 8.95 (dd, *J* =  9.0; 5.5 Hz, 1H, H5), 9.31 (d, *J* =  9.0 Hz, 1H, H4), 9.87 (d, *J* =  5.5 Hz, 1H, H6). ^13 ^C NMR (125 MHz DMSO-d_6_) *δ* 55.8 (2 × OMe), 70.7 (C7), 106.3 (C10, C14), 106.6 (C12); 129.7 (C16, C20), 129.8 (C17, C19), 130.6 (C15), 134.8 (C4), 135.2 (C9), 136.4 (C5), 137.8 (C18), 150.1 (C6), 160.2 (C3), 160.9 (C11, C13); 190.0 (C8). Anal. calcd. for C_20_H_18_BrClN_2_O_3_: C, 53.41; H, 4.03; N, 6.23%. Found: C, 53.45; H, 4.00; N, 6.27%.

*3–(4-Chlorophenyl)-1–(2-oxo-2–(3,4-dimethoxyphenyl)ethyl)pyridazin-1-ium bromide****7k***. Beige solid, Yield 77%; m.p. 144–146 °C; IR (KBr, cm^−1^): 3055, 2936, 1680, 1597, 1518, 1452, 1333, 1271, 1157, 1092, 1015. ^1^H NMR (500 MHz DMSO-d_6_) δ 3.86 (s, 3H, OMe), 3.91 (s, 3H, OMe), 6.79 (s, 2H, H7), 7.23 (d, *J* =  8.0 Hz, 1H, H13), 7.55 (s, 1H, H10), 7.76 (d, *J* =  7.5 Hz, 2H, H17, H19), 7.82 (d, *J* =  8.0 Hz, 1H, H14), 8.24 (d, *J* =  7.5 Hz, 2H, H16, H20), 8.94 (dd, *J* =  8.5; 5.5 Hz, 1H, H5), 9.30 (d, *J* =  8.5 Hz, 1H, H4), 9.87 (d, *J* =  4.5 Hz, 1H, H6). ^13 ^C NMR (125 MHz DMSO-d_6_) *δ* 55.8 (OMe), 56.0 (OMe), 70.3 (C7), 110.5 (C10), 111.3 (C13), 123.7 (C14), 126.0 (C9), 129.7 (C16, C20, C17, C19), 130.6 (C15), 134.7 (C4), 136.3 (C5), 137.8 (C18), 148.9 (C11), 150.1 (C6), 154.6 (C12); 160.1 (C3), 188.4 (C8). Anal. calcd. for C_20_H_18_BrClN_2_O_3_: C, 53.41; H, 4.03; N, 6.23%. Found: C, 53.44; H, 3.99; N, 6.27%.

*3–(4-Chlorophenyl)-1–(2-oxo-2–(4-bromophenyl)ethyl)pyridazin-1-ium bromide****7l***. Yellow solid, Yield: 75%; m.p. 200–202 °C; IR (KBr, cm^−1^): 3055, 2992, 1690, 1587, 1447, 1395, 1333, 1233, 1096, 986, 824. ^1^H NMR (500 MHz DMSO-d_6_) δ 6.86 (s, 2H, H7), 7.76 (d, *J* =  6.5 Hz, 2H, H17, H19), 7.90 (d, *J* =  6.0 Hz, 2H, H11, H13), 8.04 (d, *J* =  6.0 Hz, 2H, H10, H14), 8.25 (d, *J* =  6.5 Hz, 2H, H16, H20), 8.96 (bs, 1H, H5), 9.33 (d, *J* =  7.5 Hz, 1H, H4), 9.93 (bs, 1H, H6). ^13 ^C NMR (125 MHz DMSO-d_6_) *δ* 70.5 (C7), 129.2 (C12), 129.7 (C16, C20), 129.8 (C17, C19), 130.4 (C11, C13), 130.6 (C15), 132.3 (C10, C14), 132.4 (C9), 134.8 (C4), 136.4 (C5), 137.8 (C18), 150.1 (C6), 160.1 (C3), 189.7 (C8). Anal. calcd. for C_18_H_13_Br_2_ClN_2_O: C, 46.14; H, 2.80; N, 5.98%. Found: C, 46.14; H, 2.77; N, 6.01%.

*3–(4-Bromophenyl)-1–(2-oxo-2–(3,4,5-trimethoxyphenyl)ethyl)pyridazin-1-ium bromide****7m***. Brown solid, Yield: 75%; m.p. 185–187 °C; IR (KBr, cm^−1^): 2930, 1680, 1585, 1456, 1340, 1132. ^1^H NMR (500 MHz DMSO-d_6_) δ 3.82 (s, 3H, OMe), 3.92 (s, 6H, 2 × OMe), 6.90 (s, 2H, H7), 7.43 (s, 2H, H10, H14), 7.91 (d, *J* =  8.0 Hz, 2H, H17, H19), 8.18 (d, *J* =  8.0 Hz, 2H, H16, H20), 8.97 (dd, *J* =  9.0; 4.5 Hz, 1H, H5), 9.33 (d, *J* =  9.0 Hz, 1H, H4), 9.92 (d, *J* =  4.5 Hz, 1H, H6). ^13 ^C NMR (125 MHz DMSO-d_6_) *δ* 56.4 (2 × OMe), 60.4 (OMe), 70.6 (C7), 106.2 (C10, C14), 126.8 (C18), 128.5 (C9), 129.9 (C16, C20), 130.9 (C15), 132.6 (C17, C19), 134.7 (C4), 136.4 (C5), 143.3 (C12), 150.1 (C6), 153.0 (C11, C13), 160.3 (C3), 189.2 (C8). Anal. calcd. for C_21_H_20_Br_2_N_2_O_4_: C, 48.12; H, 3.85; N, 5.34%. Found: C, 48.15; H, 3.79; N, 5.37%.

*3–(4-Bromophenyl)-1–(2-oxo-2–(3,5-dimethoxyphenyl)ethyl)pyridazin-1-ium bromide****7n***. Brown solid, Yield: 73%; m.p. 217–220 °C; IR (KBr, cm^−1^): 3078, 2926, 1695, 1591, 1452, 1385, 1103, 1074. ^1^H NMR (500 MHz DMSO-d_6_) δ 3.85 (s, 6H, 2 × OMe), 6.82 (s, 2H, H7), 7.23 (s, 2H, H10, H14), 7.91 (d, *J* =  8.0 Hz, 2H, H17, H19), 8.17 (d, *J* =  8.0 Hz, 2H, H16, H20), 8.95 (dd, *J* =  9.0; 4.5 Hz, 1H, H5), 9.30 (d, *J* =  9.0 Hz, 1H, H4), 9.86 (d, *J* =  4.5 Hz, 1H, H6). ^13 ^C NMR (125 MHz DMSO-d_6_) *δ* 56.7 (2 × OMe), 70.7 (C7), 106.6 (C12), 107.1 (C10, C14), 127.7 (C18), 128.9 (C16, C20), 130.8 (C15), 132.7 (C17, C19), 134.8 (C4), 135.2 (C9), 136.3 (C5), 150.8 (C6), 160.3 (C3), 160.9 (C11, C13), 189.9 (C8). Anal. calcd. for C_20_H_18_Br_2_N_2_O_3_: C, 48.61; H, 3.67; N, 5.67%. Found: C, 48.65; H, 3.66; N, 5.67%.

*3–(4-Bromophenyl)-1–(2-oxo-2–(3,4-dimethoxyphenyl)ethyl)pyridazin-1-ium bromide****7o***. Brown solid, Yield: 72%; m.p. 210–212 °C; IR (KBr, cm^−1^): 3049, 2975, 1697, 1596, 1453, 1358, 1261, 1207. ^1^H NMR (500 MHz DMSO-d_6_) δ 3.86 (s, 3H, 2 OMe), 3.91 (s, 3H, 2 OMe), 6.80 (s, 2H, H7), 7.23 (d, *J* =  8.5 Hz, 1H, H13), 7.55 (d, *J* =  2.0 Hz, 1H, H10), 7.83 (dd, *J* =  8.5; 2.0 Hz, 1H, H14), 7.91 (d, *J* =  8.5 Hz, 2H, H17, H19), 8.17 (d, *J* =  8.5 Hz, 2H, H16, H20), 8.95 (dd, *J* =  9.0; 5.5 Hz, 1H, H5), 9.31 (d, *J* =  9.0 Hz, 1H, H4), 9.87 (d, *J* =  5.5 Hz, 1H, H6). ^13 ^C NMR (125 MHz DMSO-d_6_) *δ* 55.8 (OMe), 56.1 (OMe), 70.4 (C7), 110.4 (C10), 111.3 (C13), 123.7 (C14), 126.0 (C9), 126.9 (C18), 129.9 (C16, C20), 131.0 (C15), 132.7 (C17, C19), 134.7 (C4), 136.4 (C5), 148.9 (C11), 150.1 (C6), 154.6 (C12); 160.3 (C3), 188.5 (C8). Anal. calcd. for C_20_H_18_Br_2_N_2_O_3_: C, 48.61; H, 3.67; N, 5.67%. Found: C, 48.62; H, 3.64; N, 5.68%.

*3–(4-Bromophenyl)-1–(2-oxo-2–(4-bromophenyl)ethyl)pyridazin-1-ium bromide****7p***. Brown solid, Yield: 80%; m.p. 206–208 °C; IR (KBr, cm^−1^): 3055, 1690, 1587, 1447, 1387, 1333, 1233, 1076, 986, 827. ^1^H NMR (500 MHz DMSO-d_6_) δ 6.84 (s, 2H, H7), 7.90 (d, *J* =  8.5 Hz, 2H, H11, H13), 7.91 (d, *J* =  8.0 Hz, 2H, H17, H19), 8.04 (d, *J* =  8.0 Hz, 2H, H16, H20), 8.17 (d, *J* =  8.5 Hz, 2H, H10, H14), 8.95 (dd, *J* =  8.0; 5.5 Hz, 1H, H5), 9.32 (d, *J* =  8.0 Hz, 1H, H4), 9.89 (d, *J* =  5.5 Hz, 1H, H6). ^13 ^C NMR (125 MHz DMSO-d_6_) *δ* 70.5 (C7), 126.9 (C18), 129.2 (C12), 129.9 (C10, C14), 130.4 (C16, C20), 131.0 (C15), 132.2 (C11, C13), 132.4 (C9), 132.6 (C17, C19), 134.8 (C4), 136.4 (C5), 150.1 (C6), 160.3 (C3), 189.6 (C8). Anal. calcd. for C_18_H_13_Br_3_N_2_O: C, 42.14; H, 2.55; N, 5.46%. Found: C, 42.12; H, 2.54; N, 5.48%.

*1–(2-Oxo-2–(3,4,5-trimethoxyphenyl)ethyl)-3-(p-tolyl)pyridazin-1-ium bromide****7q***. Beige solid, Yield: 78%; m.p. 208–210 °C; IR (KBr, cm^−1^): 3040, 2990, 2928, 1688, 1587, 1454, 1343, 1132, 990. ^1^H NMR (500 MHz DMSO-d_6_) δ 2.43 (s, 3H, Me), 3.81 (s, 3H, OMe), 3.91 (s, 6H, 2 × OMe), 6.87 (s, 2H, H7), 7.42 (s, 2H, H10, H14), 7.48 (d, *J* =  8.0 Hz, 2H, H17, H19), 8.13 (d, *J* =  8.0 Hz, 2H, H16, H20), 8.92 (dd, *J* =  9.0; 5.5 Hz, 1H, H5), 9.28 (d, *J* =  9.0 Hz, 1H, H4), 9.86 (d, *J* =  5.5 Hz, 1H, H6). ^13 ^C NMR (125 MHz DMSO-d_6_) *δ* 21.1 (Me), 56.4 (2 × OMe), 60.4 (OMe), 70.6 (C7), 106.2 (C10, C14), 127.8 (C16, C20), 128.5 (C9), 128.9 (C15), 130.2 (C17, C19), 134.3 (C4), 136.2 (C5), 143.2 (C18), 143.3 (C12), 149.6 (C6), 153.1 (C11, C13), 161.1 (C3), 189.3 (C8). Anal. calcd. for C_22_H_23_BrN_2_O_4_: C, 57.53; H, 5.05; N, 6.10%. Found: C, 57.55; H, 5.01; N, 6.13%.

*1–(2-Oxo-2–(3,5-dimethoxyphenyl)ethyl)-3-(p-tolyl)pyridazin-1-ium bromide****7r***. Beige solid, Yield: 75%; m.p. 180–182 °C; IR (KBr, cm^−1^): 3042, 2974, 1695, 1591, 1456, 1202, 1155, 808. ^1^H NMR (500 MHz DMSO-d_6_) δ 2.43 (s, 3H, Me), 3.86 (s, 6H, 2 × OMe), 6.82 (s, 2H, H7), 6.94 (t, *J* =  2.5 Hz, 1H, H12), 7.24 (d, J = 2.5 Hz, 2H, H10, H14), 7.48 (d, *J* =  8.5 Hz, 2H, H17, H19), 8.13 (d, *J* =  8.5 Hz, 2H, H16, H20), 8.90 (dd, *J* =  9.0; 5.5 Hz, 1H, H5), 9.27 (d, *J* =  9.0 Hz, 1H, H4), 9.84 (d, *J* =  5.5 Hz, 1H, H6). ^13 ^C NMR (125 MHz DMSO-d_6_) *δ* 21.1 (Me), 55.8 (2 × OMe), 70.7 (C7), 106.3 (C10, C14), 106.6 (C12); 127.8 (C16, C20), 128.9 (C15), 130.2 (C17, C19), 134.3 (C4), 135.2 (C9), 136.1 (C5), 143.2 (C18), 149.6 (C6), 160.9 (C11, C13); 161.1 (C3), 190.1 (C8). Anal. calcd. for C_21_H_21_BrN_2_O_3_: C, 58.75; H, 4.93; N, 6.53%. Found: C, 58.77; H, 4.89; N, 6.55%.

*1–(2-Oxo-2–(3,4-dimethoxyphenyl)ethyl)-3-(p-tolyl)pyridazin-1-ium bromide****7s***. Beige solid, Yield: 75%; m.p. 150–151 °C; IR (KBr, cm^−1^): 3009, 2962, 1679, 1590, 1518, 1455, 1268, 1160, 1024. ^1^H NMR (500 MHz DMSO-d_6_) δ 2.42 (s, 3H, Me), 3.86 (s, 3H, OMe), 3.91 (s, 3H, OMe), 6.79 (s, 2H, H7), 6.94 (d, *J* =  8.5 Hz, 1H, H13), 7.55 (d, *J* =  1.5 Hz, 2H, H10), 7.48 (d, *J* =  8.0 Hz, 2H, H17, H19), 7.83 (dd, *J* =  8.5; 1.5 Hz, 1H, H14), 8.13 (d, *J* =  8.0 Hz, 2H, H16, H20), 8.90 (dd, *J* =  8.5; 5.5 Hz, 1H, H5), 9.27 (d, *J* =  9.0 Hz, 1H, H4), 9.83 (d, *J* =  5.5 Hz, 1H, H6). ^13 ^C NMR (125 MHz DMSO-d_6_) *δ* 21.1 (Me), 55.8 (OMe), 56.1 (OMe), 70.3 (C7), 110.4 (C10), 111.3 (C13); 123.7 (C14), 126.1 (C9), 127.9 (C16, C20), 128.9 (C15), 130.3 (C17, C19), 134.3 (C4), 136.1 (C5), 143.3 (C18), 148.9 (C11), 149.7 (C6), 154.5 (C12), 161.1 (C3), 188.6 (C8). Anal. calcd. for C_21_H_21_BrN_2_O_3_: C, 58.75; H, 4.93; N, 6.53%. Found: C, 58.78; H, 4.90; N, 6.56%.

*1–(2-Oxo-2–(4-bromophenyl)ethyl)-3-(p-tolyl)pyridazin-1-ium bromide****7t***. Yellow solid, Yield: 77%; m.p. 208–210 °C; IR (KBr, cm^−1^): 3022, 2988, 1694, 1584, 1452, 1219, 984. ^1^H NMR (500 MHz DMSO-d_6_) δ 2.42 (s, 3H, Me), 6.84 (s, 2H, H7), 7.47 (d, *J* =  8.0 Hz, 2H, H11, H13), 7.91 (d, *J* =  8.5 Hz, 2H, H17, H19), 8.05 (d, *J* =  8.5 Hz, 2H, H16, H20), 8.13 (d, *J* =  8.0 Hz, 2H, H10, H14), 8.91 (dd, *J* =  9.0; 6.0 Hz, 1H, H5), 9.28 (d, *J* =  9.0 Hz, 1H, H4), 9.86 (d, *J* =  6.0 Hz, 1H, H6). ^13 ^C NMR (125 MHz DMSO-d_6_) *δ* 21.1 (Me), 70.5 (C7), 127.9 (C16, C20), 128.9 (C15), 129.2 (C12), 130.2 (C17, C19), 130.4 (C11, C13), 132.3 (C10, C14), 132.5 (C9), 134.3 (C4), 136.1 (C5), 143.2 (C18), 149.7 (C6), 161.0 (C3), 189.7 (C8). Anal. calcd. for C_19_H_16_Br_2_N_2_O: C, 50.92; H, 3.60; N, 6.25%. Found: C, 50.93; H, 3.56; N, 6.26%.

*Ethyl 7–(3,4,5-trimethoxybenzoyl)pyrrolo[1,2-b]pyridazine-5-carboxylate****8a***. Beige powder, Yield: 55%; m.p. 188–190 °C; IR (KBr, cm^−1^): 1703, 1635, 1584, 1474, 1323, 1217, 1128, 1051; ^1^H NMR (500 MHz CDCl_3_) δ 1.41 (t, *J* =  7.0 Hz, 3H, CH3), 3.90 (s, 6H, 2 × OMe), 3.96 (s, 3H, OMe), 4.40 (q, *J* =  7.0 Hz, 2H, CH2), 7.15 (dd, *J* =  9.0; 4.5 Hz, 1H, H3), 7.17 (s, 2H, H12, H16), 7.77 (s, 1H, H6), 8.53 (as, 1H, H4), 8.67 (d, *J* =  9.0 Hz, 1H,H2). ^13 ^C NMR (125 MHz CDCl_3_) *δ* 14.7 (CH_3_), 55.5 (2 × OMe), 60.6 (CH_2_), 61.2 (OMe), 105.5 (C5), 107.4 (C12, C16), 117.6 (C3), 124.4 (C6), 126.8 (C7), 128.2 (C2), 133.5 (C8), 134.2 (C14), 142.2 (C11), 144.3 (C4), 153.2 (C13, C15), 163.7 (COO), 183.6 (C10). Anal. calcd. for C_20_H_20_N_2_O_6_: C, 62.49; H, 5.24; N, 7.29%. Found: C, 64.59; H, 5.15; N, 7.32%.

*Ethyl 7–(3,5-dimethoxybenzoyl)pyrrolo[1,2-b]pyridazine-5-carboxylate****8b***. Yellow powder, Yield: 57%; m.p. 178–180 °C; IR (KBr, cm^−1^): 1685, 1631, 1587, 1479, 1358, 1225, 1047 (C-O); ^1^H NMR (500 MHz CDCl_3_) δ 1.40 (t, *J* =  7.0 Hz, 3H, CH_3_), 3.85 (s, 6H, 2 × OMe), 4.39 (q, *J* =  7.0 Hz, 2H, CH_2_), 6.68 (s, 1H, H14), 7.01 (d, *J* =  1.5 Hz, 2H, H12, H16), 7.15 (dd, *J* =  9.5; 4.5 Hz, 1H, H3), 7.78 (s, 1H, H6), 8.54 (d, *J* =  3.0 Hz, 1H, H4), 8.67 (d, *J* =  9.0 Hz, 1H, H2). ^13 ^C NMR (125 MHz CDCl_3_) *δ* 14.6 (CH_3_), 55.8 (2 × OMe), 60.6 (CH_2_), 104.7 (C14), 105.5 (C5), 107.6 (C12, C16), 117.8 (C3), 125.2 (C6), 126.7 (C7), 128.2 (C2), 133.7 (C8), 141.1 (C11), 144.4 (C4), 160.8 (C13, C15), 163.7 (COO), 184.1 (C10). Anal. calcd. for C_19_H_18_N_2_O_5_: C, 64.40; H, 5.12; N, 7.91%. Found: C, 64.52; H, 5.05; N, 7.99%.

*Ethyl 7–(3,4-dimethoxybenzoyl)pyrrolo[1,2-b]pyridazine-5-carboxylate****8c***. Beige solid, Yield: 50%; m.p. 123–124 °C; IR (KBr, cm^−1^): 1704, 1629, 1580, 1371, 1132, 1023; ^1^H NMR (500 MHz CDCl_3_) δ 1.41 (t, *J* =  7.0 Hz, 3H, CH_3_), 3.96 (s, 3H, OMe), 3.98 (s, 3H, OMe), 4.39 (q, *J* =  7.0 Hz, 2H, CH_2_), 6.95 (d, 1H, *J* =  8.0 Hz, H15), 7.12 (dd, *J* =  9.5; 4.5 Hz, 1H, H3), 7.55 (d, *J* =  1.5 Hz, 1H, H12), 7.56 (dd, *J* =  8.0; 1.5 Hz, 1H, H16), 7.75 (s, 1H, H6), 8.50 (dd, *J* =  4.0; 2.0 Hz, 1H, H4), 8.65 (dd, *J* =  9.0; 2.0 Hz, 1H, H2). ^13 ^C NMR (125 MHz CDCl_3_) *δ* 14.7 (CH_3_), 56.2 (OMe), 56.3 (OMe), 60.5 (CH_2_), 105.2 (C5), 110.1 (C12), 112.0 (C15), 117.3 (C3), 123.9 (C16), 124.8 (C6), 126.9 (C7), 128.2 (C2), 131.7 (C11), 133.2 (C8), 144.2 (C4), 149.2 (C13), 153.2 (C14), 163.8 (COO), 184.4 (C10). Anal. calcd. for C_19_H_18_N_2_O_5_: C, 64.40; H, 5.12; N, 7.91%. Found: C, 64.44; H, 5.07; N, 7.97%.

*Ethyl 7–(4-bromobenzoyl)pyrrolo[1,2-b]pyridazine-5-carboxylate****8d***. Yellow solid, Yield: 57%; m.p. 133–135 °C; IR (KBr, cm^−1^): 1707, 1638, 1468, 1235, 1190, 1096; ^1^H NMR (500 MHz CDCl_3_) δ 1.40 (t, *J* =  7.0 Hz, 3H, CH_3_), 4.38 (q, *J* =  7.0 Hz, 2H, CH_2_), 7.17 (dd, *J* =  9.0; 4.5 Hz, 1H, H3), 7.65 (d, *J* =  8.0 Hz, 2H, H13, H15), 7.72 (s, 1H, H6), 7.77 (d, *J* =  8.0 Hz, 2H, H12, H16), 8.54 (dd, *J* =  4.0; 1.5 Hz, 1H, H4), 8.67 (dd, *J* =  9.5; 4.0 Hz, 1H, H2). ^13 ^C NMR (125 MHz CDCl_3_) *δ* 14.6 (CH_3_), 60.7 (CH_2_), 105.7 (C5), 118.0 (C3), 125.0 (C6), 126.4 (C7), 127.4 (C14), 128.2 (C2), 131.2 (C12, C16), 131.9 (C13, C15), 133.8 (C8), 137.9 (C11), 144.5 (C4), 163.6 (COO), 183.3 (C10). Anal. calcd. for C_17_H_13_BrN_2_O_3_: C, 54.71; H, 3.51; N, 7.51%. Found: C, 54.74; H, 3.47; N, 7.57%.

*Ethyl 2-methyl-7–(3,4,5-trimethoxybenzoyl)pyrrolo[1,2-b]pyridazine-5-carboxylate****8e***. Beige solid; Yield: 47%; m.p. 91–93 °C; IR (KBr, cm^−1^): 2978,1695, 1656, 1586, 1470, 1368, 1233, 1124, 754; ^1^H NMR (500 MHz CDCl_3_) δ 1.40 (t, *J* =  7.0 Hz, 3H, CH_3_), 2.64 (s, 3H,Me), 3.90 (s, 6H, 2 × OMe), 3.95 (s, 3H, OMe), 4.38 (q, *J* =  7.0 Hz, 2H, CH_2_), 7.02 (d, *J* =  9.0 Hz, 1H, H3), 7.18 (s, 2H, H12, H16), 7.68 (s, 1H, H6), 8.52 (d, *J* =  9.0 Hz, 1H, H4). ^13 ^C NMR (125 MHz CDCl_3_) *δ* 14.7 (CH_3_), 22.4 (Me), 56.5 (2 × OMe), 60.5 (CH_2_), 61.2 (OMe), 105.1 (C5), 107.5 (C12, C16), 119.7 (C3), 123.8 (C6), 126.6 (C7), 127.6 (C11), 132.3 (C8), 134.3 (C14), 142.2 (C4), 153.1 (C13, C15), 153.4 (C2), 163.9 (COO), 183.5 (C10). Anal. calcd. for C_21_H_22_N_2_O_6_: C, 63.31; H, 5.57; N, 7.03%. Found: C, 63.29; H, 5.55; N, 7.00%.

*Ethyl 2-methyl-7–(3,5-dimethoxybenzoyl)pyrrolo[1,2-b]pyridazine-5-carboxylate****8f***. Beige solid, Yield: 45%; m.p. 96–98 °C; IR (KBr, cm^−1^): 2937, 1681, 1654, 1592, 1436, 1361, 1232, 1158, 754; ^1^H NMR (500 MHz CDCl_3_) δ 1.39 (t, *J* =  7.0 Hz, 3H, CH_3_), 2.65 (s, 3H,Me), 3.84 (s, 6H, 2 × OMe), 4.37 (q, *J* =  7.0 Hz, 2H, CH_2_), 6.68 (s, 1H, H14), 7.02 (overlapped signals, 3H, H3, H12, H16), 7.69 (s, 1H, H6), 8.52 (d, *J* =  9.5 Hz, 1H, H4). ^13 ^C NMR (125 MHz CDCl_3_) *δ* 14.6 (CH_3_), 22.4 (Me), 55.8 (2 × OMe), 60.5 (CH_2_), 104.6 (C14), 105.2 (C5), 107.6 (C12, C16), 119.9 (C3), 124.6 (C6), 126.6 (C7), 127.6 (C11), 132.5 (C8), 141.2 (C4), 153.5 (C2), 160.7 (C13, C15), 163.8 (COO), 184.1 (C10). Anal. calcd. for C_20_H_20_N_2_O_5_: C, 65.21; H, 5.47; N, 7.60%. Found: C, 65.18; H, 5.45; N, 7.63%.

*Ethyl 2-methyl-7–(4-bromobenzoyl)pyrrolo[1,2-b]pyridazine-5-carboxylate****8h***. Beige solid, Yield: 42%; m.p. 145–147 ˚C; IR (KBr, cm^−1^): 2990, 1701, 1645, 1547, 1462, 1362, 1261, 1229, 1188, 1098, 754; ^1^H NMR (500 MHz CDCl_3_) δ 1.40 (t, *J* =  7.0 Hz, 3H, CH_3_), 2.65 (s, 3H, Me), 4.38 (q, *J* =  7.0 Hz, 2H, CH_2_), 7.04 (d, *J* =  9.5 Hz, 1H, H3), 7.63 (s, 1H, H6), 7.65 (d, *J* =  8.0 Hz, 2H, H13, H15), 7.77 (d, *J* =  8.0 Hz, 2H, H12, H16), 8.54 (d, *J* =  9.0 Hz, 1H, H4). ^13 ^C NMR (125 MHz CDCl_3_) *δ* 14.6 (CH_3_), 22.4 (Me), 60.6 (CH_2_), 105.4 (C5), 120.1 (C3), 124.4 (C6), 126.3 (C7), 127.3 (C14), 127.6 (C4), 131.2 (C12, C16), 131.8 (C13, C15), 132.6 (C8), 138.1 (C11), 153.7 (C2), 163.7 (COO), 183.3 (C10). Anal. calcd. for C_18_H_15_BrN_2_O_3_: C, 55.83; H, 3.90; N, 7.23%. Found: C, 55.85; H, 3.87; N, 7.26%.

*Ethyl 2–(4-chlorophenyl)-7–(3,4,5-trimethoxybenzoyl)pyrrolo[1,2-b]pyridazine-5-carboxylate****8i***. Beige solid, Yield: 41%; m.p. 230–232 ˚C; IR (KBr, cm^−1^): 2984, 1697, 1657, 1583, 1503, 1460, 1314, 1234, 1169, 1130, 808; ^1^H NMR (500 MHz CDCl_3_) δ 1.42 (t, *J* =  7.0 Hz, 3H, CH_3_), 3.90 (s, 6H, 2 × OMe), 3.97 (s, 3H, OMe), 4.42 (q, *J* =  7.0 Hz, 2H, CH_2_), 7.20 (s, 2H, H12, H16), 7.47 (d, *J* =  8.5 Hz, 2H, H19, H21), 7.47 (d, *J* =  9.0 Hz, 1H, H4), 7.79 (s, 1H, H6), 8.01 (d, *J* =  8.5 Hz, 2H, H18, H22), 8.69 (d, *J* =  9.0 Hz, 1H, H3). ^13 ^C NMR (125 MHz CDCl_3_) *δ* 14.7 (CH_3_), 56.5 (2 × OMe), 60.6 (CH_2_), 61.2 (OMe), 105.6 (C5), 107.3 (C12, C16), 115.8 (C4), 124.4 (C6), 127.1 (C7), 128.4 (C3, C18, C22), 129.5 (C19, C21), 132.2 (C11), 133.6 (C17), 134.2 (C8), 136.9 (C20), 142.3 (C14), 151.1 (C2), 153.2 (C13, C15), 163.7 (COO), 183.6 (C10). Anal. calcd. for C_26_H_23_ClN_2_O_6_: C, 63.10; H, 4.68; N, 5.66%. Found: C, 63.15; H, 4.67; N, 5.69%.

*Ethyl 2–(4-chlorophenyl)-7–(3,5-dimethoxybenzoyl)pyrrolo[1,2-b]pyridazine-5-carboxylate****8j***. Beige solid, Yield: 41%; m.p. 193–195 ˚C; IR (KBr, cm^−1^): 2949, 1694, 1659, 1599, 1458, 1298, 1094, 810; ^1^H NMR (500 MHz CDCl_3_) δ 1.42 (t, *J* =  7.0 Hz, 3H, CH_3_), 3.85 (s, 6H, 2 × OMe), 4.40 (q, *J* =  7.0 Hz, 2H, CH_2_), 6.71 (bs, 1H, H14), 7.05 (d, *J* =  2.0 Hz, 2H, H12, H16), 7.47 (d, *J* =  8.5 Hz, 2H, H19, H21), 7.59 (d, *J* =  9.5 Hz, 1H4), 7.81 (s, 1H, H6), 8.01 (d, *J* =  8.5 Hz, 2H, H18, H22), 8.70 (d, *J* =  9.5 Hz, 1H, H3). ^13 ^C NMR (125 MHz CDCl_3_) *δ* 14.7 (CH_3_), 55.8 (2 × OMe), 60.6 (CH_2_), 104.8 (C14), 105.7 (C5), 107.5 (C12, C16), 115.9 (C4), 125.0 (C6), 127.1 (C7), 128.4 (C18, C22), 128.5 (C3), 129.5 (C19, C21), 132.4 (C8), 133.7 (C17), 136.8 (C20), 141.1 (C11), 151.2 (C2), 160.9 (C13, C15), 163.7 (COO), 184.2 (C10). Anal.calcd. for C_25_H_21_ClN_2_O_5_: C, 64.59; H, 4.55; N, 6.03%. Found: C, 64.64; H, 4.50; N, 6.10%.

*Ethyl 2–(4-chlorophenyl)-7–(3,4-dimethoxybenzoyl)pyrrolo[1,2-b]pyridazine-5-carboxylate****8k***. Beige solid, Yield: 42%; m.p. 161–163 ˚C; IR (KBr, cm^−1^): 2982, 1696, 1649, 1595, 1462, 1269, 1234, 1090, 806; ^1^H NMR (500 MHz CDCl_3_) δ 1.43 (t, *J* =  7.0 Hz, 3H, CH_3_), 3.97 (s, 3H, OMe), 3.99 (s, 3H, OMe), 4.41 (q, *J* =  7.0 Hz, 2H, CH_2_), 6.95 (d, *J* =  8.0 Hz, 1H, H15), 7.46 (d, *J* =  8.5 Hz, 2H, H19, H21), 7.56 (m, 3H, H12, H16, H4), 7.76 (s, 1H, H6), 8.00 (d, *J* =  8.5 Hz, 2H, H18, H22), 8.68 (d, *J* =  9.5 Hz, 1H, H3). ^13 ^C NMR (125 MHz CDCl_3_) *δ* 14.8 (CH_3_), 56.3 (OMe), 56.4 (OMe), 60.7 (CH_2_), 105.4 (C5), 110.2 (C15), 112.0 (C12), 115.7 (C4), 124.2 (C6), 124.9 (C16), 127.4 (C7), 128.6 (C3, C18, C22), 129.5 (C19, C21), 131.9 (C11), 132.1 (C17), 133.8 (C8), 136.9 (C20), 151.1 (C2), 149.4 (C13), 153.4 (C14), 163.9 (COO), 183.5 (C10). Anal. calcd. for C_25_H_21_ClN_2_O_5_: C, 64.59; H, 4.55; N, 6.03%. Found: C, 64.60; H, 4.49; N, 6.07%.

*Ethyl 2–(4-chlorophenyl)-7–(4-bromobenzoyl)pyrrolo[1,2-b]pyridazine-5-carboxylate****8l***. White solid; Yield: 45%; m.p. 163–165 ˚C; IR (KBr, cm^−1^) 3051, 2988, 1694, 1458, 1242, 1207, 1090, 808; ^1^H N,R (500 MHz CDCl_3_) δ 1.43 (t, *J* =  7.0 Hz, 3H, CH_3_), 4.41 (q, *J* =  7.0 Hz, 2H, CH_2_), 7.47 (d, *J* =  8.5 Hz, 2H, H19, H21), 7.59 (d, *J* =  9.5 Hz, H4), 7.67 (d, *J* =  8.0 Hz, 2H, H12, H16), 7.76 (s, 1H, H6), 7.78 (d, *J* =  8.0 Hz, 2H, H13, H15), 7.96 (d, *J* =  8.5 Hz, 2H, H18, H22), 8.70 (d, *J* =  9.5 Hz, 1H, H3). ^13 ^C NMR (125 MHz CDCl_3_) *δ* 14.7 (CH_3_), 60.7 (CH_2_), 105.9 (C5), 116.1 (C4), 124.8 (C6), 126.9 (C14), 127.4 (C7), 128.4 (C18, C22), 128.5 (C3), 129.5 (C19, C21), 132.4 (C8), 133.5 (C17), 131.1 (C13, C15), 131.9 (C12, C16), 137.0 (C20), 138.0 (C11), 151.3 (C2), 163.6 (COO), 183.5 (C10). Anal. calcd. for C_23_H_16_BrClN_2_O_3_: C, 57.11; H, 3.33; N, 5.79%. Found: C, 57.10; H, 3.29; N, 5.85%.

*Ethyl 2–(4-bromophenyl)-7–(3,4,5-trimethoxybenzoyl)pyrrolo[1,2-b]pyridazine-5-carboxylate****8m***. Beige solid, Yield: 50%; m.p. 238–240 ˚C; IR (KBr, cm^−1^): 2984, 2930, 1697, 1657, 1586, 1503, 1458, 1314, 1234, 1169, 1128, 808, 752; ^1^H NMR (500 MHz CDCl_3_) δ 1.42 (t, *J* =  7.0 Hz, 3H, CH_3_), 3.90 (s, 6H, 2 × OMe), 3.97 (s, 3H, OMe), 4.42 (q, *J* =  7.0 Hz, 2H, CH_2_), 7.20 (s, 2H, H12, H16), 7.58 (d, *J* =  9.0 Hz, H4), 7.63 (d, *J* =  8.5 Hz, 2H, H19, H21), 7.80 (s, 1H, H6), 7.94 (d, *J* =  8.5 Hz, 2H, H18, H22), 8.69 (d, *J* =  9.0 Hz, 1H, H3). ^13 ^C NMR (125 MHz CDCl_3_) *δ* 14.7 (CH_3_), 56.5 (2 × OMe), 60.6 (CH_2_), 61.2 (OMe), 105.6 (C5), 107.4 (C12, C16), 115.7 (C4), 124.4 (C6), 125.3 (C20), 127.1 (C7), 128.5 (C3), 128.7 (C18, C22), 132.5 (C19, C21), 132.2 (C11), 134.1 (C17), 134.2 (C8), 142.3 (C14), 151.2 (C2), 153.2 (C13, C15), 163.7 (COO), 183.6 (C10). Anal. calcd. for C_26_H_23_BrN_2_O_6_: C, 57.90; H, 4.30; N, 5.19%. Found: C, 57.95; H, 4.27; N, 5.24%.

*Ethyl 2–(4-bromophenyl)-7–(3,5-dimethoxybenzoyl)pyrrolo[1,2-b]pyridazine-5-carboxylate****8n***. Beige solid, Yield: 51%; m.p. 193–195 ˚C; IR (KBr, cm^−1^): 2982, 1695, 1659, 1591, 1458, 1298, 1155, 1096, 808; ^1^H NMR (500 MHz CDCl_3_) δ 1.42 (t, *J* =  7.0 Hz, 3H, CH_3_), 3.85 (s, 6H, 2 × OMe), 4.40 (q, *J* =  7.0 Hz, 2H, CH_2_), 6.71 (t, *J* =  2.5 Hz, 1H, H14), 7.05 (d, *J* =  2.5 Hz, 2H, H12, H16), 7.58 (d, *J* =  9.5 Hz, H4), 7.63 (d, *J* =  8.5 Hz, 2H, H19, H21), 7.81 (s, 1H, H6), 7.94 (d, *J* =  8.5 Hz, 2H, H18, H22), 8.70 (d, *J* =  9.5 Hz, 1H, H3). ^13 ^C NMR (125 MHz CDCl_3_) *δ* 14.7 (CH_3_), 55.8 (2 × OMe), 60.6 (CH_2_), 104.8 (C14), 105.7 (C5), 107.5 (C12, C16), 115.9 (C4), 125.0 (C6), 125.2 (C20), 127.1 (C7), 128.4 (C3), 128.7 (C18, C22), 132.4 (C19, C21, C8), 134.1 (C17), 141.1 (C11), 151.2 (C2), 160.9 (C13, C15), 163.7 (COO), 184.2 (C10). Anal. calcd. for C_25_H_21_BrN_2_O_5_: C, 58.95; H, 4.16; N, 5.50%. Found: C, 58.94; H, 4.09; N, 5.55%.

*Ethyl 2–(4-bromophenyl)-7–(3,4-dimethoxybenzoyl)pyrrolo[1,2-b]pyridazine-5-carboxylate****8o***. Beige solid, Yield: 52%; m.p. 166–167 ˚C; IR (KBr, cm^−1^): 2975, 2929, 1719, 1680, 1592, 1458, 1269, 1236, 1147, 1087; ^1^H NMR (500 MHz CDCl_3_) δ 1.42 (t, *J* =  7.0 Hz, 3H, CH_3_), 3.97 (s, 3H, OMe), 3.99 (s, 3H, OMe), 4.41 (q, *J* =  7.0 Hz, 2H, CH_2_), 6.95 (d, *J* =  7.5 Hz, 1H, H15), 7.57–7.62 (m, 5H, H12, H16, H4, H19, H21), 7.76 (s, 1H, H6), 7.93 (d, *J* =  8.0 Hz, 2H, H18, H22), 8.67 (d, *J* =  9.5 Hz, 1H, H3). ^13 ^C NMR (125 MHz CDCl_3_) *δ* 14.7 (CH_3_), 56.2 (OMe), 56.3 (OMe), 60.6 (CH_2_), 105.4 (C5), 110.1 (C15), 111.8 (C12), 115.5 (C4), 124.1 (C6), 124.8 (C16), 125.1 (C20), 127.3 (C7), 128.4 (C3), 128.7 (C18, C22), 131.7 (C11), 132.4 (C19, C21), 132.0 (C8), 134.2 (C17), 149.3 (C13), 151.1 (C2), 153.2 (C14), 163.8 (COO), 183.4 (C10). Anal. calcd. for C_25_H_21_BrN_2_O_5_: C, 58.95; H, 4.16; N, 5.50%. Found: C, 58.97; H, 4.10; N, 5.53%.

*Ethyl 2–(4-bromophenyl)-7–(4-bromobenzoyl)pyrrolo[1,2-b]pyridazine-5-carboxylate****8p***. White solid, Yield: 45%; m.p. 171–173 ˚C; IR (KBr, cm^−1^): 3051, 2988, 1694, 1657, 1458, 1242, 1209, 1094, 1072, 806, 748; ^1^H NMR (500 MHz CDCl_3_) δ 1.42 (t, *J* =  7.0 Hz, 3H, CH_3_), 4.41 (q, *J* =  7.0 Hz, 2H, CH_2_), 7.63 (d, *J* =  8.5 Hz, 2H, H19, H21), 7.59 (d, *J* =  9.5 Hz, H4), 7.67 (d, *J* =  8.0 Hz, 2H, H12, H16), 7.76 (s, 1H, H6), 7.77 (d, *J* =  8.0 Hz, 2H, H13, H15), 7.88 (d, *J* =  8.5 Hz, 2H, H18, H22), 8.70 (d, *J* =  9.5 Hz, 1H, H3). ^13 ^C NMR (125 MHz CDCl_3_) *δ* 14.6 (CH_3_), 60.7 (CH_2_), 105.9 (C5), 116.0 (C4), 124.8 (C6), 125.3 (C20), 126.9 (C14), 127.4 (C7), 128.5 (C3), 128.7 (C18, C22), 131.1 (C19, C21), 131.9 (C13, C15), 132.4 (C8), 132.5 (C12, C16), 133.9 (C17), 138.0 (C11), 151.4 (C2), 163.6 (COO), 183.5 (C10). Anal. calcd. for C_23_H_16_Br_2_N_2_O_3_: C, 52.30; H, 3.05; N, 5.30%. Found: C, 52.30; H, 3.00; N, 5.32%.

*Ethyl 2-(p-tolyl)-7–(3,4,5-trimethoxybenzoyl)pyrrolo[1,2-b]pyridazine-5-carboxylate****8q***. White solid, Yield: 40%; m.p. 198–200 ˚C; IR (KBr, cm^−1^): 3020, 2978, 2943, 1697, 1657, 1586, 1503, 1460, 1333, 1234, 1130, 1130, 806, 750; ^1^H NMR (500 MHz CDCl_3_) δ 1.42 (t, *J* =  7.0 Hz, 3H, CH_3_), 2.41 (s, 3H, Me), 3.89 (s, 6H, 2 × OMe), 3.97 (s, 3H, OMe), 4.41 (q, *J* =  7.0 Hz, 2H, CH_2_), 7.20 (s, 2H, H12, H16), 7.29 (d, *J* =  8.5 Hz, 2H, H19, H21), 7.60 (d, *J* =  9.5 Hz, 1H4), 7.78 (s, 1H, H6), 7.94 (d, *J* =  8.5 Hz, 2H, H18, H22), 8.66 (d, *J* =  9.5 Hz, 1H, H3). ^13 ^C NMR (125 MHz CDCl_3_) *δ* 14.7 (CH_3_), 21.5 (Me), 56.5 (2 × OMe), 60.5 (CH_2_), 61.2 (OMe), 105.4 (C5), 107.3 (C12, C16), 116.3 (C4), 124.2 (C6), 127.0 (C18, C22), 127.1 (C7), 128.1 (C3), 129.9 (C19, C21), 132.2 (C11), 132.3 (C17), 134.3 (C8), 140.9 (C20), 142.1 (C14), 152.2 (C2), 153.1 (C13, C15), 163.8 (COO), 183.7 (C10). Anal. calcd. for C_27_H_26_N_2_O_6_: C, 68.34; H, 5.52; N, 5.90%. Found: C, 68.35; H, 5.47; N, 5.94%.

*Ethyl 2-(p-tolyl)-7–(3,5-dimethoxybenzoyl)pyrrolo[1,2-b]pyridazine-5-carboxylate****8r***. Beige solid, Yield: 40%; m.p. 142–144 ˚C; IR (KBr, cm^−1^): 2999, 2918, 1686, 1649, 1593, 1452, 1302, 1236, 1159, 1053, 804, 754; ^1^H NMR (500 MHz CDCl_3_) δ 1.42 (t, *J* =  7.0 Hz, 3H, CH_3_), 2.41 (s, 3H, Me), 3.84 (s, 6H, 2 × OMe), 4.40 (q, *J* =  7.0 Hz, 2H, CH_2_), 6.97 (bs, 1H, H14), 7.05 (bs, 2H, H12, H16), 7.29 (d, *J* =  8.0 Hz, 2H, H19, H21), 7.60 (d, *J* =  9.5 Hz, H4), 7.79 (s, 1H, H6), 7.95 (d, *J* =  8.5 Hz, 2H, H18, H22), 8.65 (d, *J* =  9.5 Hz, 1H, H3). ^13 ^C NMR (125 MHz CDCl_3_) *δ* 14.7 (CH_3_), 21.5 (Me), 55.8 (2 × OMe), 60.5 (CH_2_), 104.7 (C14), 105.4 (C5), 107.4 (C12, C16), 116.4 (C4), 124.9 (C6), 127.0 (C7), 127.1 (C18, C22), 128.0 (C3), 129.9 (C19, C21), 132.3 (C8), 132.5 (C17), 140.8 (C20), 141.3 (C11), 152.3 (C2), 160.8 (C13, C15), 163.8 (COO), 184.2 (C10). Anal. calcd. for C_26_H_24_N_2_O_5_: C, 70.26; H, 5.44; N, 6.30%. Found: C, 70.29; H, 5.39; N, 6.33%.

*Ethyl 2-(p-tolyl)-7–(3,4-dimethoxybenzoyl)pyrrolo[1,2-b]pyridazine-5-carboxylate****8s***. Beige solid, Yield: 40%; m.p. 150–151 ˚C; IR (KBr, cm^−1^): 3088, 2974, 2929, 1721, 1681, 1614, 1514, 1457, 1272, 1148, 1088; ^1^H NMR (500 MHz CDCl_3_) δ 1.42 (t, *J* =  7.0 Hz, 3H, CH_3_), 3.96 (s, 3H, OMe), 3.98 (s, 3H, OMe), 4.41 (q, *J* =  7.0 Hz, 2H, CH_2_), 6.95 (d, *J* =  8.0 Hz, 1H, H15), 7.28 (d, *J* =  8.0 Hz, 2H, H19, H21), 7.59 (m, 3H, H12, H16, H4), 7.74 (s, 1H, H6), 7.94 (d, *J* =  8.0 Hz, 2H, H18, H22), 8.65 (d, *J* =  9.5 Hz, 1H, H3). ^13 ^C NMR (125 MHz CDCl_3_) *δ* 14.7 (CH_3_), 56.2 (OMe), 56.3 (OMe), 60.5 (CH_2_), 105.0 (C5), 110.0 (C15), 111.8 (C12), 116.1 (C4), 123.9 (C6), 124.7 (C16), 127.1 (C18, C22), 127.2 (C7), 128.0 (C3), 129.9 (C19, C21), 131.9 (C11), 132.1 (C17), 132.4 (C8), 140.7 (C20), 149.2 (C13), 152.1 (C2), 153.1 (C14), 164.0 (COO), 183.5 (C10). Anal. calcd. for C_26_H_24_N_2_O_5_: C, 70.26; H, 5.44; N, 6.30%. Found: C, 70.30; H, 5.40; N, 6.35%.

*Ethyl 2-(p-tolyl)-7–(4-bromobenzoyl)pyrrolo[1,2-b]pyridazine-5-carboxylate****8t***. Yellow solid, Yield: 40%; m.p. 160–162 ˚C; IR (KBr, cm^−1^): 3072, 2974, 1697, 1620, 1503, 1452, 1219, 1211, 1082, 816; ^1^H NMR (500 MHz CDCl_3_) δ 1.42 (t, *J* =  7.0 Hz, 3H, CH_3_), 2.42 (s, 3H, Me), 4.41 (q, *J* =  7.0 Hz, 2H, CH_2_), 7.29 (d, *J* =  8.5 Hz, 2H, H19, H21), 7.62 (d, *J* =  9.5 Hz, H4), 7.67 (d, *J* =  7.5 Hz, 2H, H12, H16), 7.75 (s, 1H, H6), 7.76 (d, *J* =  8.0 Hz, 2H, H13, H15), 7.89 (d, *J* =  8.0 Hz, 2H, H18, H22), 8.67 (d, *J* =  9.5 Hz, 1H, H3). ^13 ^C NMR (125 MHz CDCl_3_) *δ* 14.7 (CH_3_), 21.6 (Me), 60.6 (CH_2_), 105.7 (C5), 116.5 (C4), 124.6 (C6), 127.0 (C14), 127.1 (C18, C22), 127.2 (C7), 128.1 (C3), 130.0 (C19, C21), 132.2 (C17), 132.5 (C8), 131.1 (C13, C15), 131.9 (C12, C16), 138.0 (C11), 141.0 (C20), 152.4 (C2), 163.7 (COO), 183.6 (C10). Anal. calcd. for C_24_H_19_BrN_2_O_3_: C, 62.22; H, 4.13; N, 6.05%. Found: C, 62.25; H, 4.05; N, 6.08%.

*2–(2-Oxo-2–(3,4,5-trimethoxyphenyl)ethyl)phthalazin-2-ium bromide****10a***. Brown solid, Yield: 77%; m.p. 150–152 ˚C; IR (KBr, cm^−1^): 2976, 1670, 1584, 1339, 1125, 764. ^1^H NMR (500 MHz DMSO-d_6_) δ 3.80 (s, 3H, OMe), 3.91 (s, 6H, 2 × OMe), 6.87 (s, 2H, H11), 7.44 (s, 2H, H14, H18), 8.50 (t, *J* =  7.5 Hz, 1H, H6), 8.62 (t, *J* =  8.0 Hz, H5), 8.69 (d, *J* =  8.0 Hz, 1H, H4), 8.76 (d, *J* =  8.0 Hz, 1H, H7), 10.24 (s, 1H, H3), 10.77 (s, 1H, H8). ^13 ^C NMR (125 MHz DMSO-d_6_) *δ* 56.4 (2 × OMe), 60.4 (OMe), 69.0 (C11), 106.3 (C14, C18), 127.3 (C10), 127.5 (C9), 128.6 (C13), 128.7 (C4), 130.8 (C7), 136.6 (C6), 139.9 (C5), 143.3 (C16), 153.1 (C15, C17), 153.5 (C8), 154.9 (C3), 189.6 (C12). Anal. calcd. for C_19_H_19_BrN_2_O_4_: C, 54.43; H, 4.57; N, 6.68%. Found: C, 54.47; H, 4.54; N, 6.71%.

*2–(2-Oxo-2–(3,5-dimethoxyphenyl)ethyl)phthalazin-2-ium bromide****10b***. Brown solid, Yield: 76%; m.p. 178–180 ˚C; IR (KBr, cm^−1^): 2976, 1703, 1589, 1319, 1011. ^1^H NMR (500 MHz DMSO-d_6_) δ 3.86 (s, 6H, 2 × Me), 6.79 (s, 2H, H11), 6.93 (t, *J* =  2.0 Hz, 1H, H16), 7.26 (d, *J* =  2.0 Hz, 2H, H14, H18), 8.51 (t, *J* =  8.0 Hz, 1H, H6), 8.61 (t, *J* =  8.0 Hz, H5), 8.67 (d, *J* =  8.0 Hz, 1H, H4), 8.75 (d, *J* =  8.0 Hz, 1H, H7), 10.21 (s, 1H, H3), 10.69 (s, 1H, H8). ^13 ^C NMR (125 MHz DMSO-d_6_) *δ* 55.8 (2 × OMe), 69.1 (C11), 106.3 (C14, C18), 106.6 (C16), 127.3 (C10), 127.5 (C9), 128.6 (C4), 130.8 (C7), 135.3 (C13), 136.6 (C6), 139.9 (C5), 153.6 (C8), 154.9 (C3), 160.9 (C15, C17), 190.5 (C12). Anal. calcd. for C_18_H_17_BrN_2_O_3_: C, 55.54; H, 4.40; N, 7.20%. Found: C, 55.55; H, 4.37; N, 7.22%.

*2–(2-Oxo-2–(3,4-dimethoxyphenyl)ethyl)phthalazin-2-ium bromide****10c***. Brown solid, Yield: 74%; m.p. 224–226 ˚C; IR (KBr, cm^−1^): 3017, 2974, 1701, 1589, 1313, 1204, 1011. ^1^H NMR (500 MHz DMSO-d_6_) δ 3.86 (s, 3H, OMe), 3.92 (s, 3H, OMe), 6.74 (s, 2H, H11), 6.98 (d, *J* =  8.0 Hz, 1H, H17), 7.47 (overlapped signals, 2H, H14, H18), 8.47 (t, *J* =  8.0 Hz, 1H, H6), 8.60 (t, *J* =  8.0 Hz, H5), 8.68 (d, *J* =  8.0 Hz, 1H, H4), 8.76 (d, *J* =  8.0 Hz, 1H, H7), 10.25 (s, 1H, H3), 10.72 (s, 1H, H8). ^13 ^C NMR (125 MHz DMSO-d_6_) *δ* 55.8 (OMe), 56.1 (OMe), 68.9 (C11), 110.5 (C14), 111.4 (C18), 123.8 (C17), 126.2 (C13), 127.5 (C9), 128.7 (C4), 130.9 (C7), 136.6 (C6), 139.9 (C5), 148.9 (C15), 153.5 (C8), 154.9 (C3), 154.5 (C16), 188.9 (C12). Anal. calcd. for C_18_H_17_BrN_2_O_3_: C, 55.54; H, 4.40; N, 7.20%. Found: C, 55.55; H, 4.37; N, 7.22%.

*2–(2-Oxo-2–(4-bromophenyl)ethyl)phthalazin-2-ium bromide****10d***. White solid, Yield: 76%; m.p. 222–224 ˚C; IR (KBr, cm^−1^): 3013, 1684, 1613, 1580, 1352. ^1^H NMR (500 MHz DMSO-d_6_) δ 6.83 (s, 2H, H_11_) 7.89 (d, *J* = 8.5 Hz, 2H, H15, H17), 8.08 (d, *J* =  8.5 Hz, 2H, H14, H18), 8.48 (t, *J* =  8.0 Hz, 1H, H6), 8.61 (t, *J* =  8.0 Hz, H5), 8.68 (d, *J* =  8.0 Hz, 1H, H4), 8.75 (d, *J* =  8.0 Hz, 1H, H7), 10.23 (s, 1H, H3), 10.76 (s, 1H, H8). ^13 ^C NMR (125 MHz DMSO-d_6_) *δ* 68.8 (C11), 127.3 (C10), 127.5 (C9), 128.6 (C4), 129.2 (C16), 130.5 (C14, C18), 130.8 (C7), 132.3 (C15, C17), 132.5 (C13), 136.6 (C6), 139.9 (C5), 153.5 (C8), 154.9 (C3), 190.1 (C12). Anal. calcd. for C_16_H_12_Br_2_N_2_O: C, 47.09; H, 2.96; N, 6.86%. Found: C, 48.11; H, 2.94; N, 6.88%.

*Ethyl 3–(3,4,5-trimethoxybenzoyl)pyrrolo[2,1-a]phthalazine-1-carboxylate****11a***. White solid, Yield: 40%; m.p. 235–237 ˚C; IR (KBr, cm^−1^): 3041, 2933, 2835, 1711, 1650, 1583, 1261, 1172, 1130, 1042, 785; ^1^H NMR (500 MHz CDCl_3_) δ 1.41 (t, *J* =  7.0 Hz, 3H, CH_3_), 3.90 (s, 6H, 2 × OMe), 3.90 (s, 3H, OMe), 4.41 (q, *J* =  7.0 Hz, 2H, CH_2_), 7.24 (s, 2H, H16, H20), 7.73 (s, 1H, H2), 7.76 (d, *J* =  7.5 Hz, 1H, H7), 7.90 (overlapped signals, 2H, H8, H6), 8.75 (s, 1H, H5), 9.84 (d, *J* =  8.0 Hz, 1H, H9). ^13 ^C NMR (125 MHz CDCl_3_) *δ* 14.6 (CH_3_), 56.5 (2 × OMe), 60.9 (CH_2_), 61.2 (OMe), 107.6 (C16, C20), 108.3 (C1), 122.3 (C11), 124.2 (C2), 127.0; 127.1 (C12, C3), 127.6 (C9), 127.7 (C6), 129.8 (C7), 130.2 (C13), 133.1 (C8), 134.1 (C15), 142.3 (C18), 146.5 (C5), 153.1 (C17, C19), 164.4 (COO), 183.8 (C14). Anal. calcd. for C_24_H_22_N_2_O_6_: C, 66.35; H, 5.10; N, 6.45%. Found: C, 66.58; H, 5.05; N, 6.48%.

*Ethyl 3–(3,5-dimethoxybenzoyl)pyrrolo[2,1-a]phthalazine-1-carboxylate****11b***. White solid, Yield: 41%; m.p. 220–222 ˚C; IR (KBr, cm^−1^): 3040, 2969, 2835, 1707, 1649, 1595, 1458, 1383, 1173, 1096, 756; ^1^H NMR (500 MHz CDCl_3_) δ 1.41 (t, *J* =  7.0 Hz, 3H, CH_3_), 3.85 (s, 6H, 2 × OMe), 4.41 (q, *J* =  7.0 Hz, 2H, CH_2_), 6.70 (bs, 1H, H18), 7.08 (d, *J* =  2.0 Hz, 2H, H16, H20), 7.74 (overlapped signals, 2H, H2, H7), 7.90 (overlapped signals, 2H, H8, H6), 8.76 (s, 1H, H5), 9.83 (d, *J* =  8.5 Hz, 1H, H9). ^13 ^C NMR (125 MHz CDCl_3_) *δ* 14.6 (CH_3_), 55.8 (2 × OMe), 60.9 (CH_2_), 105.0 (C18), 107.8 (C16, C20), 108.4 (C1), 122.3 (C11), 124.8 (C2), 127.0; 127.1 (C12, C3), 127.6 (C9), 127.7 (C6), 129.8 (C7), 130.4 (C13), 133.0 (C8), 141.1 (C15), 146.6 (C5), 160.7 (C17, C19), 164.4 (COO), 184.3 (C14). Anal. calcd. for C_23_H_20_N_2_O_5_: C, 68.31; H, 4.98; N, 6.93%. Found: C, 68.34; H, 4.95; N, 6.96%.

*Ethyl 3–(3,4-dimethoxybenzoyl)pyrrolo[2,1-a]phthalazine-1-carboxylate****11c***. White solid, Yield: 41%; m.p. 226–228 ˚C; IR (KBr, cm^−1^): 3101, 2959, 2932, 1713, 1645, 1466, 1412, 1263, 1177, 1024, 764; ^1^H NMR (500 MHz CDCl_3_) δ 1.41 (t, *J* =  7.0 Hz, 3H, CH_3_), 3.96 (s, 3H, OMe), 3.97 (s, 3H, OMe), 4.40 (q, *J* =  7.0 Hz, 2H, CH_2_), 6.94 (d, *J* =  8.0 Hz, 1H, H19), 7.58 (overlapped signals, 2H, H16, H20), 7.68 (s, 1H, H2), 7.73 (t, *J* =  7.5 Hz, 1H, H7), 7.88 (overlapped signals, 2H, H8, H6), 8.71 (s, 1H, H5), 9.82 (d, *J* =  8.0 Hz, 1H, H9). ^13 ^C NMR (125 MHz CDCl_3_) *δ* 14.6 (CH_3_), 56.22 (OMe), 56.23 (OMe), 60.8 (CH_2_), 108.1 (C1), 110.1 (C16), 112.1 (C19), 122.2 (C11), 123.5 (C2), 125.1 (C20), 127.1 (C3), 127.3 (C12), 127.5 (C9), 127.7 (C6), 129.7 (C7), 129.9 (C13), 131.7 (C15), 133.0 (C8), 146.4 (C5), 149.2 (C17), 153.3 (C18), 164.5 (COO), 183.8 (C14). Anal. calcd. for C_23_H_20_N_2_O_5_: C, 68.31; H, 4.98; N, 6.93%. Found: C, 68.32; H, 4.97; N, 6.94%.

*Ethyl 3–(4-bromobenzoyl)pyrrolo[2,1-a]phthalazine-1-carboxylate****11d***^35^. White solid, Yield: 51%; m.p. 180–182 ˚C; IR (KBr, cm^−1^): 2980, 1722, 1653, 1587, 1464, 1379, 1242; ^1^H NMR (500 MHz CDCl_3_) δ 1.38 (t, *J* =  7.0 Hz, 3H, CH_3_), 4.37 (q, *J* =  7.0 Hz, 2H, CH_2_), 7.62 (d, *J* =  8.5 Hz, 2H, H17, H19), 7.65 (s, 1H, H2), 7.74 (t, *J* =  8.0 Hz, 1H, H7), 7.78 (d, *J* =  8.5 Hz, 2H, H16, H20), 7.87 (overlapped signals, 2H, H8, H6), 8.73 (s, 1H, H5), 9.80 (d, *J* =  8.5 Hz, 1H, H9). ^13 ^C NMR (125 MHz CDCl_3_) *δ* 14.5 (CH_3_), 60.9 (CH_2_), 108.5 (C1), 122.3 (C11), 124.7 (C2), 126.7 (C12); 126.8 (C18), 127.5 (C3), 127.6 (C9), 127.7 (C6), 129.9 (C7), 130.5 (C13), 131.3 (C16, C20), 131.7 (C17, C19), 133.1 (C8), 137.9 (C15), 146.6 (C5), 164.2 (COO), 183.5 (C14). Anal. calcd. for C_21_H_15_BrN_2_O_3_: C, 59.59; H, 3.57; N, 6.62%. Found: C, 59.62; H, 3.55; N, 6.64%.

### Molecular modelling

Flexible docking experiments were carried out in Autodock Vina^40^, using a 18x22x22 Å3 grid box centered on the colchicine binding site of the α,β-tubulin heterodimer crystal structure (PDB: 1SA0)^41^. The 3 D structures of the compounds were constructed in Avogadro v1.2.0^42^ and were subjected to 10,000 steepest descent steps of energy minimisation in the MMFF94 force field. One hundred poses were generated for each ligand, and the best-ranked models were chosen for further visual inspection in order to assess the consistency of the generated docking solutions relative to the docking poses of known inhibitor colchicine. Molecular graphics and visual analyses were performed in The PyMOL Molecular Graphics System, Version 1.8.2. (Schrödinger, LLC). Log*p* values were calculated using the ChemAxon/Chemicalize server (www.chemicalize.com).

### Cell proliferation assay

The compounds were tested against a panel of 60 human cancer cell lines at the National Cancer Institute, Rockville, MD. The cytotoxicity experiments were realised using a 48 h exposure protocol using sulphorhodamine B assay^43–45^.

## Results and discussion

### Chemistry

The chosen method for the assembly of pyrrolo[1,2-*b*]pyridazine moieties relied on 1,3-dipolar cycloaddition of different pyridazinium ylides to ethyl propiolate.

**Scheme 1:**
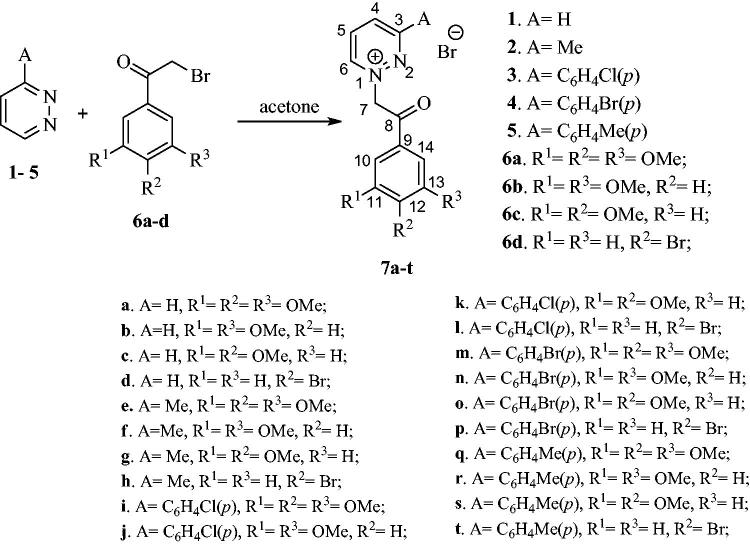
Synthesis of pyridazin-1-ium quaternary salts **7a–t.**

First, pyridazines **1**–**5** (Scheme 1) were used for the synthesis of their monoquaternary salts with 2-bromoacetophenones **6a–d**. While compound **6d** is commercially available, compounds **6a–c** were synthesised using reported procedures^46^. The quaternisation reactions were carried out at room temperature (r.t.) in a minimal amount of acetone, leading to the formation of salts **7a–t** (Scheme 1).

**Scheme 2:**
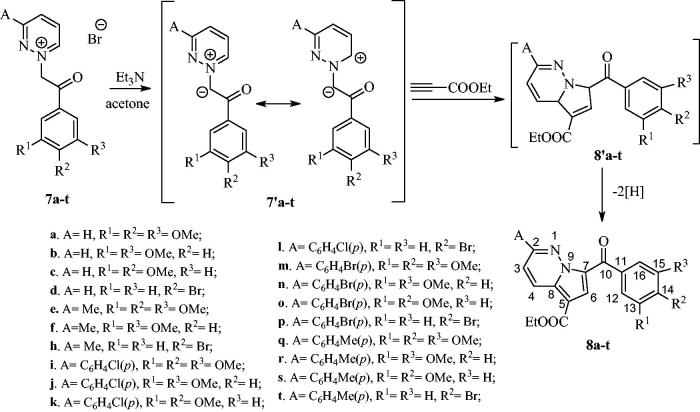
Synthesis of pyrrolo[1,2-*b*]pyridazines **8a–t.**

As shown in Scheme 2, ethyl propiolate was reacted with the corresponding pyridazinium ylides **7′a–t** (*in situ* generated in basic medium from salts **7a–t**) to give the intermediate dihydropyrrolo[1,2-*b*]pyridazines **8′a–t**, which in turn underwent oxidative dehydrogenation under atmospheric conditions, yielding the final compounds **8a**–**t** in moderate yields (40–52%) (Scheme 2).

**Scheme 3:**
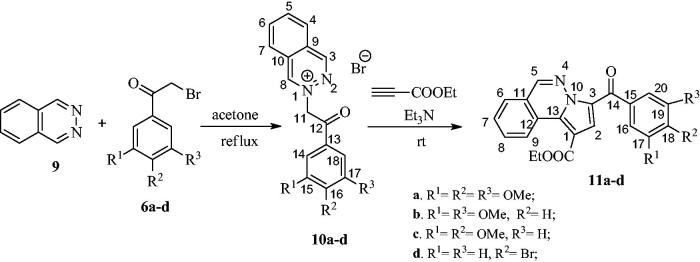
Synthesis of pyrrolo[2,1-*a*]phthalazines **11a–d** from phthalazine via quaternary phthalazinum salts **10a–d.**

For the synthesis of compounds **11**, in a similar manner, phthalazine was first reacted with 2-bromoacetophenones **6a–d**, to give monoquaternary salts **10a**–**d** (Scheme 3). Phthalazinium salts **10a–d** furnished pyrrolo[2,1-*a*]phthalazines **11a–d** when treated with triethylamine and ethyl propiolate in acetone at room temperature (Scheme 3).

### Biological activity

Fourteen of the synthesised compounds (**8a, b, d, e, f, h, i, j, k, n, q,** and **11a–c**) were selected by the National Cancer Institute (NCI) for screening against a panel of 60 human tumour cell lines at a single dose of 10 μM^43^, the representative results for the active compounds being summarised in Table 1.

**Table 1.** Results of the *in vitro* growth inhibition (GI %) caused by compounds **8a, b, d, e, f, h** and **11a-c** against human cancer cell lines in the single-dose assay^a^.

Cell type	Compound	8a	8b	8d	8e	8f	8h	11a	11b	11c
	Cell line	GI (%) (10^−5^M)^a^								
Leukemia	CCRF-CEM	89	76	23	83	86	4	30	77	11
	K-562	89	78	79	**90**	**90**	6	77	**94**	11
	SR	82	68	72	77	82	18	77	**95**	27
	HL-60(TB)	**100^b^ (33)**	**100^b^ (22)**	47	**100^b^ (24)**	**100^b^ (17)**	11	71	**100^b^ (8)**	18
	MOLT-4	81	67	31	82	70	23	38	71	31
	RPMI-8226	79	71	51	82	88	22	18	67	21
Non-small Cell lung cancer	A549/ATCC	76	65	27	73	73	23	45	82	20
	HOP-62	68	69	23	49	52	21	10	57	10
	NCI-H460	**90**	85	0	**90**	89	0	49	**93**	0
	NCI-H522	65	75	84	**96**	**100^b^ (11)**	34	60	**97**	16
Colon cancer	COLO205	**100^b^ (25)**	**100^b^ (13)**	29	**100^b^ (39)**	**100^b^ (11)**	1	40	81	0
	HCT-116	88	86	54	85	75	22	49	**96**	19
	HCT-15	76	78	30	75	68	8	41	70	23
	HT-29	**96**	**93**	40	**92**	**96**	27	70	**98**	0
	SW-620	66	81	60	73	82	6	64	**92**	1
	KM12	79	72	48	72	70	8	60	84	4
CNS cancer	SF-295	86	72	26	59	76	1	27	**91**	13
	SF-539	**100^b^ (12)**	80	22	85	**96**	6	13	89	4
	SNB-75	66	76	31	52	82	21	21	**100^b^ (10)**	14
	U251	85	75	35	81	77	28	19	**100^b^ (13)**	14
Melanoma	LOX IMVI	57	60	9	69	51	11	34	**90**	4
	M14	**96**	**100^b^ (16)**	45	79	89	0	50	84	6
	MDA-MB-435	**100^b^ (57)**	**99**	88	**97**	**100^b^ (16)**	0	93	**96**	1
	UACC-62	70	74	39	41	53	7	30	58	1
	SK-MEL-2	42	79	55	72	84	19	22	85	3
	SK-MEL-5	74	74	37	**97**	69	10	66	81	34
Ovarian cancer	OVCAR-3	**100^b^ (15)**	**99**	22	87	**99**	0	53	**100^b^ (13)**	2
	NCI/ADR-RES	**97**	**93**	50	79	81	7	36	81	12
	SK-OV-3	73	80	27	76	**94**	25	17	62	7
	OVCAR-8	70	62	24	75	70	10	22	66	12
	OVCAR-4	38	38		48	43	19	16	**100^b^ (18)**	0
Renal cancer	A498	**100^b^ (5)**	**100^b^ (3)**	15	77	**100^b^ (2)**	7	26	84	18
	ACHN	48	47		51	40	1	21	**98**	9
	RXF393	**100^b^ (4)**	64	25	66	86	21	12	71	10
	TK-10	46	36		22	35	20	3	**100^b^ (9)**	15
Breast cancer	MCF7	80	75	51	78	79	12	70	84	14
	MDA-MB-468	**100^b^ (12)**	73	22	63	70	8	1	57	5
Prostate cancer	PC-3	78	64	33	69	75	24	45	68	24
	DU-145	76	63	6	79	78	16	6	54	2

The most active compounds are highlighted in bold.

a Data obtained from NCI’s *in vitro* 60 cell one dose screening at 10^−5^M concentration.

b Cytotoxic effect; lethality percent is represented in brackets.

Pyrrolo[2,1-*b*]pyridazines **8a, 8b, 8e, 8f,** and pyrrolo[2,1-*a*]phthalazine **11b** showed a very good growth inhibition effect on almost all 60 cell lines, the best results being registered on leukemia HL-60 (TB) cell, colon cancer COLO205 cell, melanoma MDA-MB-435 cell, ovarian cancer cell OVCAR-3, and renal cancer A498 cell. Compound **8a** also showed a moderate cytotoxic effect, notably on melanoma MDA-MB-435 cells (57% cytotoxic). Mild to moderate cytotoxic effects were also observed for compounds **8b**, **8e**, **8f,** and **11b** against several cell types. Interestingly, the substitution of pyrrolo[2,1-*b*]pyridazine heterocycle at position 2 with a 4-substituted phenyl group resulted in the loss of the activity, compounds **8i–j**, **8k**, **8n,** and **8q** showing almost no inhibition effect against the 60 cell tested lines (data not shown). In contrast, 2-methylpyrrolo[2,1-*b*]pyridazines **8e–f** showed similar activity to unsubstituted compounds **8a–b**. Another interesting aspect is that substitution of the 3,4,5-trimethoxyphenyl ring of with a 3,5-dimethoxyphenyl one, did not diminish the inhibitory activity. In fact, pyrrolo[2,1-*a*]phthalazine compound **11b** showed better growth inhibitory properties when compared with **11a**. Substitution of the 3,4,5-trimethoxyphenyl ring with 3,4-dimethoxyphenyl or 4-bromophenyl also caused a reduction in biological activity, with the exception of compound **8d**, which maintained moderate GI% values on most tested cell lines, although lower than 3,4,5-trimethoxyphenyl-substituted analogues **8a–b**.

The most active compounds **8a, 8b, 8e, 8f,** and **11b** were selected for the second stage five dose-response studies^42–44^ selected results being presented in Table 2.

**Table 2.** Results of the 5-dose *in vitro* human cancer cell growth inhibition^a^ for compounds **8a–b, e–f** and **11b** and compared with standard drug Doxorubicin.

Cell type	Compound →	8a	8b	8e	8f	11b	Doxorubicin^c^
	Cell line ↓	GI_50_ (nM)^b^					
Leukemia	CCRF-CEM	261	2510	212	348	n.d.	79
	HL-60(TB)	228	1380	160	248	820	126
	K-562	**90.6**	538	n.d.	n.d.	n.d.	200
	MOLT-4	443	2630	396	527	n.d.	32
	RPMI-8226	246	1820	n.d.	n.d.	n.d.	79
	SR	**46.1**	573	**48.1**	**75.9**	442	25
Non-small Cell Lung cancer	A549/ATCC	487	11100	223	767	n.d.	63
	HOP-62	363	10200	398	691	n.d.	63
	NCI-H460	312	2580	133	365	494	16
	NCI-H522	343	346	171	303	236	32
Colon cancer	COLO205	193	797	n.d.	n.d.	n.d.	200
	HCT-116	276	n.d.	164	331	455	79
	HCT-15	171	587	**84.4**	280	484	6310
	HT-29	208	403	133	401	384	126
	KM12	216	n.d.	**57.7**	254	351	251
	SW-620	155	518	**68.7**	280	483	100
CNS cancer	SF-268	733	26500	550	676	1590	100
	SF-295	180	2100	**65.7**	311	483	100
	SF-539	276	1850	130	349	1060	126
	SNB-19	769	4260	420	752	1570	40
	SNB-75	211	399	n.d.	384	471	63
	U251	402	2000	331	549	730	40
Melanoma	MALME-3M	247	>100000	n.d.	n.d.	1070	126
	M14	176	394	136	251	485	159
	MDA-MB-435	**31.4**	221	**25.6**	**45.6**	188	251
	SK-MEL-2	385	738	495	494	n.d.	159
	SK-MEL-5	269	508	**58.5**	276	623	79
	UACC-62	176	523	**61.5**	477	692	159
Ovarian cancer	OVCAR-3	145	402	**64.9**	289	341	398
	NCI/ADR-RES	200	463	123	308	476	7943
	SK-OV-3	426	4900	546	878	n.d.	200
Renal cancer	786-0	395	11400	335	523	n.d.	126
	A498	**46.6**	n.d.	**76.2**	388	n.d.	100
	CAKI-1	301	2976	n.d.	n.d.	n.d.	1000
	RXF 393	185	1640	116	239	1070	100
Prostate cancer	PC-3	166	7450	**93.9**	317	839	316
	DU-145	333	3960	391	906	n.d.	100
Breast cancer	MCF7	**94.6**	1310	**48.1**	313	410	40
	HS 578T	236	1990	190	284	1840	316
	BT-549	437	1220	878	1990	1180	251
	T-47D	n.d.^c^	17200	>100000	501	n.d.	63
	MDA-MB-468	281	1110	**66.8**	297	403	50

The most active compounds are highlighted in bold.

a Data obtained from NCI’s *in vitro* 60 cell 5-dose screening^43–45^.

b GI_50_ – the molar concentration of tested compound causing 50% growth inhibition of tumor cells. Determined at five concentration levels (100, 10, 1.0, 0.1 and 0.01 μM).

c GI_50_ data for Doxorubicin tested at a highest concentration of 100 μM were obtained from NCI database: https://dtp.cancer.gov/dtpstandard/dwindex/index.jsp.

n.d.: Not determined.

All five tested compounds confirmed the preliminary results by displaying good antiproliferative properties. The best candidate, 2-methyl-pyrrolo[2,1-b]pyridazine **8e** exhibited GI_50_ values <100 nM in thirteen cell lines, notably on melanoma MDA-MB-435 cell (GI_50_ = 25.6 nM), leukemia SR cell (GI_50_ = 48.1 nM) and breast cancer MCF7 cell (GI_50_ = 48.1 nM). Compound **8e** showed better GI_50_ values against melanoma MDA-MB-435, SK-MEL-5, and UACC-62 and colon cancer HCT-15, KM12 and SW-620 cell than Doxorubicin (NSC: 123127 code), the NCI standard drug for this type of analysis.

Interestingly, even if it exhibits an overall inhibitory activity lower than the 2-methyl substituted compound **8e**, compound **8a** shows an excellent inhibitory activity on melanoma MDA-MB-435 cell (GI_50_ = 31.4 nM), leukemia SR cell (GI_50_ = 46.1 nM) and renal cancer A498 (GI_50_ = 46.6 nM). Also, compound **8f** displayed very good activity against melanoma MDA-MB-435 cell (GI_50_ = 45.6 nM).

Notably, compound **8e** showed a very good cytostatic activity on melanoma MDA-MB-435 cell with a total growth inhibition level of effect (TGI) of 76.3 nM and leukemia HL-60(TB) (TGI = 588 nM), whereas compounds **8a** and **8f** showed the best cytostatic activity on melanoma MDA-MB-435 cell with a TGI of 117 nM and 420 nM, respectively. Significant cytotoxic activity was exhibited only by compound **8a** on MDA-MB-435 melanoma cell with a lethal concentration (LC_50_) value of 438 nM.

Although pyrrolo[2,1-a]phthalazine **11b** displayed the best mean growth inhibitory effect in preliminary single dose evaluation (Table 1), it did not exhibit GI_50_ values under the 100 nm threshold, as was the case for the more simple pyrrolo[1,2-b]pyridazines. Therefore, the introduction of a bulkier heterocycle, such as pyrrolo[2,1-a]phthalazine, in place of the 3′-hydroxy-4′-methoxyphenyl ring of phenstatin is less favourable in terms of antiproliferative activity than pyrrolo[1,2-b]pyridazine.

### Molecular modelling

Because both computational and biological models of 3,4,5-trimethoxyphenyl-containing phenstatin analogues supported the hypothesis that the antiproliferative effects of these compounds are induced by inhibiting tubulin polymerisation^5^^,^^7^^,^^33^^,^^47^^,^^48^, docking experiments were performed on the colchicine binding site of the α,β-tubulin heterodimer (PDB:1SA0), in order to evaluate the shape and electrostatic complementarity between ligands and the α,β-tubulin heterodimer interface, which could account for the observed antiproliferative effects.

**Figure 2:**
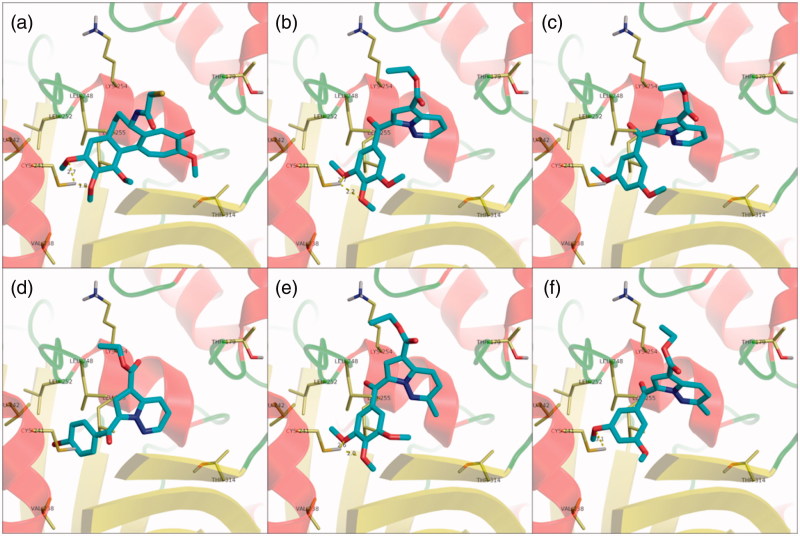
Structure and docking of diazines in the tubulin binding site: (a) DAMA-colchicine, (b) **8a**, (c) **8b**, (d) **8d**, (e) **8e**, (f) **8f**; the α,β-tubulin heterodimer is represented as ribbons; amino acids in the binding site are represented as sticks.

Compounds **8a** and **8b** displayed similar docking conformations grouped into two distinct clusters, both having the trimethoxyphenyl subunit overlapping with the one in the co-crystallised DAMA-colchicine ligand (Figure 2(a)), and interacting with the protein through hydrogen bonding with βCys241. The ligands are further stabilised in the binding pocket through hydrophobic interactions with βLeu242, βLeu248, βAla250, βLeu252, βLeu255, and βVal238. The diazine moiety either extended on top of the binding pocket, with the ester functional group orienting towards the dimer interface (Figure 2(b,c)), or was flipped at about 180°, to have the ester group roughly overlapping with the third colchicine ring in the crystal structure. Interestingly, the 4-bromo-substituted compound **8d**, which displayed a less pronounced biological activity than **8a** and **8b**, adopted a conformation in which the *p*-bromo substituted phenyl was accommodated more deeply in the colchicine binding pocket, resulting in a shift in the position of the central heterocyclic moiety towards the center of the colchicine binding site, which led to the disruption of the hydrogen bond with βCys241 (Figure 2(d)).

Overall, the docking experiments suggest that the removal of the 4-methoxy group does not influence the accommodation of the ligand in the binding pocket, in agreement with the biological data, while the introduction of a bromine atom as substituent can induce a different binding conformation which leads to the disruption of the hydrogen bond between the ligand and βCys241, which could account for the reduced antiproliferative activity of compound **8d**.

**Figure 3:**
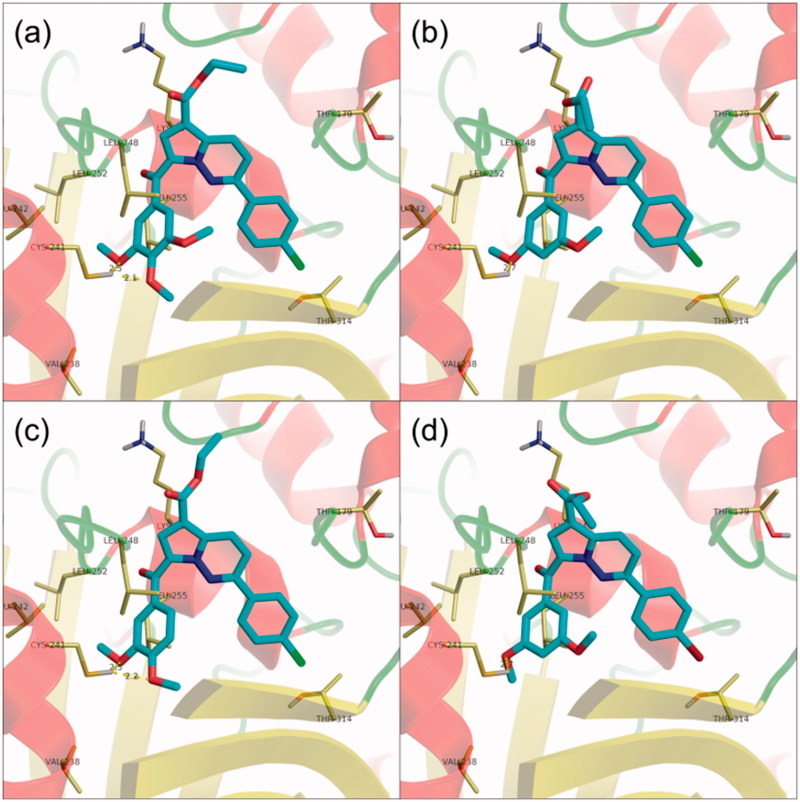
Structure and docking of diazines in the tubulin binding site: (a) **8i**, (b) **8j**, (c) **8k**, (d) **8n**; the α,β-tubulin heterodimer is represented as ribbons; amino acids in the binding site are represented as sticks.

The 2-methyl-substituted analogues of **8a** and **8b** (**8e** and **8f**) were accommodated in a similar fashion to that of parent compounds (Figure 3(e,f)), suggesting that the introduction of a methyl substituent does not influence the binding preferences of the compounds in the colchicine binding site, in agreement with the biological data in terms of antiproliferative activity. Compound **8h**, which displayed a marked reduction in biological activity when compared to parent compound **8d**, did not form the two expected well-defined clusters of conformations, but rather had a broad range of unrelated docking poses, the most energetically favourable being similar to the second cluster of compound **8d**.

Interestingly, 2-(*p-*halogeno-phenyl)-substituted compounds **8i, 8j, 8k,** and **8n**, which showed a marked decrease in growth inhibition activity when compared to unsubstituted analogues, were compatible with the colchicine binding site, and were accommodated in a similar fashion to their unsubstituted or 2-methyl-substituted analogues with biological activity to the α,β-tubulin heterodimer (Figures 2 and 3). A closer inspection of the basic physicochemical properties of these four compounds reveals, however, a violation of Lipinski’s rule of five in terms of log*p* values^49^ (Table 3), which could account for the loss in antiproliferative efficacy in spite of apparent activity at the colchicine binding site^50^.

**Table 3.** Theoretical log*p* values of biologically tested compounds.

Compound	log*p*	Compound	log*p*	Compound	log*p*
**8a**	2.77	**8h**	4.14	**8q**	5.32
**8b**	2.93	**8i**	5.41	**11a**	3.76
**8d**	4.01	**8j**	5.56	**11b**	3.92
**8e**	2.90	**8k**	5.56	**11c**	3.92
**8f**	3.06	**8n**	5.73		

Values were calculated using the ChemAxon/Chemicalize server (www.chemicalize.com).

**Figure 4:**
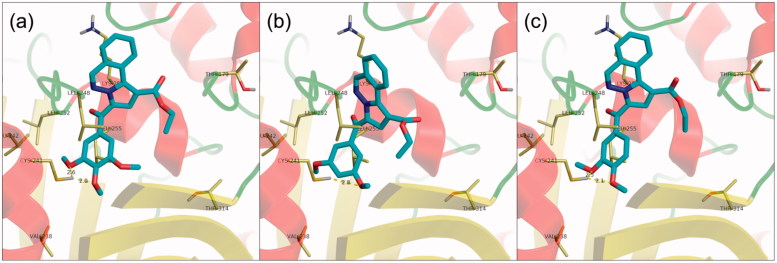
Structure and docking of diazines in the tubulin binding site: (a) **11a**, (b) **11b**, (c) **11c**; the α,β-tubulin heterodimer is represented as ribbons; amino acids in the binding site are represented as sticks.

Docking of pyrrolo[2,1-a]phthalazines **11a–c** revealed a single cluster of conformations for each compound, similar to the second cluster obtained for pyrrolo[1,2-b]pyridazines **8a**, **8b**, **8e,** and **8f**, in which the heterocyclic subunit is oriented as to have the ester group roughly overlapping with the third colchicine ring in the crystal structure (Figure 4). The methoxyphenyl subunit is stabilised by a hydrogen bond interaction with βCys241, similar to the case of pyrrolo[1,2-b]pyridazine analogues. Notably, compound **11b** adopts a conformation slightly deeper in the hydrophobic pocket, which induces a rotation of the heterocyclic core and facilitates a hydrophobic interaction with βLeu248, which is unique among the three docked pyrrolo[2,1-a]phthalazines. A tighter hydrophobic interaction between **11b** and the protein could account for the pronounced antiproliferative activity exerted by **11b** among the three tested pyrrolo[2,1-a]phthalazines.

However, for all compounds, complementary tubulin polymerisation assays are needed in order to confirm the proposed molecular mechanism.

## Conclusion

In summary, five of the newly synthesised pyrrolo[1,2-*b*]pyridazine and pyrrolo[2,1-*a*]phthalazine phenstatin analogues showed *in vitro* antiproliferative activity, the most potent being compounds **8f** with GI_50_ values <100 nM on thirteen cell lines including colon, ovarian, renal, prostate, brain and breast cancer, melanoma and leukemia. Notably, compound **8a** showed a very good antiproliferative effect on melanoma MDA-MB-435 cell, renal cancer A498, and leukemia SR cell. The substitution of position 2 of pyrrolo[1,2-*b*]pyridazine with a methyl group generally appears to increase the antiproliferative potency of the compounds, while the introduction of a more bulkier substituent is completely detrimental for the growth inhibitory properties, despite the fact that docking studies showed a good compatibility with the colchicine binding site of tubulin. The lack of proliferative activity in the case of the bulkier 2–(4-X-phenyl)-pyrrolo[1,2-*b*]pyridazines could be explained by the suboptimal lipophilicity and solubility of these compounds. However, further assaying in terms of tubulin polymerisation is needed in order to confirm the proposed antiproliferative mechanism of action of the newly synthetised compounds. Compound **8f** could serve as a useful lead compound for further structural optimisation in the development of new anticancer agents.
